# *Escherichia coli* monothiol glutaredoxin GrxD replenishes Fe-S clusters to the essential ErpA A-type carrier under low iron stress

**DOI:** 10.1016/j.jbc.2024.107506

**Published:** 2024-06-27

**Authors:** Claire E. Fisher, Daniel W. Bak, Kennedy E. Miller, Clorissa L. Washington-Hughes, Anna M. Dickfoss, Eranthie Weerapana, Béatrice Py, F. Wayne Outten

**Affiliations:** 1 Department of Chemistry and Biochemistry, University of South Carolina, Columbia, South Carolina, USA; 2 Department of Chemistry, Boston College, Massachusetts, USA; 3 Aix-Marseille Université-Centre National de la Recherche Scientifique (UMR7283), Laboratoire de Chimie Bactérienne, Institut de Microbiologie de la Méditerranée, Institut Microbiologie Bioénergies et Biotechnologie, Marseille, France

**Keywords:** iron-sulfur (Fe-S) protein, monothiol glutaredoxin GrxD, A-type carrier ErpA, mass spectrometry (MS), chemoproteomics, *E. coli*, iron metabolism, metal homeostasis

## Abstract

Iron-sulfur (Fe-S) clusters are required for essential biological pathways, including respiration and isoprenoid biosynthesis. Complex Fe-S cluster biogenesis systems have evolved to maintain an adequate supply of this critical protein cofactor. In *Escherichia coli,* two Fe-S biosynthetic systems, the “housekeeping” Isc and “stress responsive” Suf pathways, interface with a network of cluster trafficking proteins, such as ErpA, IscA, SufA, and NfuA. GrxD, a Fe-S cluster-binding monothiol glutaredoxin, also participates in Fe-S protein biogenesis in both prokaryotes and eukaryotes. Previous studies in *E. coli* showed that the Δ*grxD* mutation causes sensitivity to iron depletion, spotlighting a critical role for GrxD under conditions that disrupt Fe-S homeostasis. Here, we utilized a global chemoproteomic mass spectrometry approach to analyze the contribution of GrxD to the Fe-S proteome. Our results demonstrate that (1) GrxD is required for biogenesis of a specific subset of Fe-S proteins under iron-depleted conditions, (2) GrxD is required for cluster delivery to ErpA under iron limitation, (3) GrxD is functionally distinct from other Fe-S trafficking proteins, and (4) GrxD Fe-S cluster binding is responsive to iron limitation. All these results lead to the proposal that GrxD is required to maintain Fe-S cluster delivery to the essential trafficking protein ErpA during iron limitation conditions.

Iron-sulfur (Fe-S) clusters are protein cofactors required for key biological pathways, including respiration, amino acid and isoprenoid biosynthesis, and DNA repair (1, 2). Fe-S clusters contain iron (Fe^2/3+^) bound to sulfide (S^2-^), most commonly as [2Fe-2S], [3Fe-4S], and [4Fe-4S] clusters coordinated by protein cysteine (Cys), or less commonly, histidine (His), or aspartate (Asp) residues. Since Fe-S clusters are both essential and sensitive to disruption by reactive oxygen and nitrogen species or thiophilic metals (3, 4, 5, 6), multiple Fe-S cluster biogenesis systems have evolved to maintain cluster maturation (7, 8, 9). These include the iron-sulfur cluster (Isc) pathway (10, 11), the sulfur formation (Suf) pathway (12), the nitrogen-fixation pathway (13, 14, 15), specialized for maturation of nitrogenase, and the recently described minimalist pathways, Mis and Sms (16). In eukaryotes, the Isc pathway is conserved in mitochondria, while the Suf pathway is largely conserved in the chloroplast. The gram-negative model organism *Escherichia coli,* harbors both the Isc pathway, utilized under “housekeeping” iron-replete conditions, and the Suf pathway, activated by complex transcriptional and posttranscriptional regulation under stress conditions (such as iron starvation or oxidative stress) (17, 18). Each pathway is expressed from its own polycistronic operon, *iscRSUA*-*hscBA*-*fdx*-*iscX* or *sufABCDSE*, and encodes for a cysteine desulfurase (IscS or SufSE), a Fe-S scaffold protein (IscU or the SufBC_2_D complex), and an A-type Fe-S cluster carrier (ATC) protein (IscA or SufA). Isc also contains molecular chaperones (HscAB), an electron-donating ferredoxin (Fdx), a Fe-S cluster-binding transcriptional regulator (IscR), and IscX, whose role in Fe-S biogenesis still remains to be defined.

Nascent Fe-S clusters formed on the IscU or SufBC_2_D scaffolds enter a complex cluster trafficking network responsible for distributing clusters to downstream Fe-S protein targets. The dimeric ATC proteins are an essential part of this network. *E. coli* contains three members of the ATC family, IscA, SufA, and ErpA, which show 30% sequence identity to each other but are not fully redundant *in vivo* (19, 20). The IscA and SufA proteins are encoded in the *isc* or *suf* operons and this close genetic proximity with their scaffold proteins is a hallmark of the ATC-II subfamily (19). Both *in vitro* and *in vivo* evidence shows that IscA and SufA (and their eukaryotic homologs) can act as intermediates between their respective scaffolds and other Fe-S trafficking proteins, to mediate cluster delivery to Fe-S target proteins. Based on phylogenetic analysis, it was proposed that the ATC-I subfamily proteins, such as *E. coli* ErpA, have evolved in close partnership with their apoprotein targets rather than with a specific scaffold protein (19). This hypothesis is consistent with genetic experiments that place ErpA downstream of IscA and SufA in the trafficking network under aerobiosis, where it likely receives its Fe-S cluster from IscA or SufA before transferring it to the final apoprotein targets (19). Altogether, the studies on ATCs have shown that they form an adaptable network, where the individual contribution of each ATC varies in response to changing environmental conditions and depending on the final target. Adaptation of the ATC delivery network also involves recruitment of additional components such as NfuA in *E. coli*. NfuA interacts with ErpA and increases the stability of the ErpA-bound Fe-S cluster (21). NfuA is a two-domain protein with an N-terminal “degenerate” A-type carrier domain (ATC∗) lacking Fe-S cluster co-ordinating Cys ligands, which is fused to an Nfu domain (22, 23). While the Nfu domain binds a [4Fe-4S] cluster, the ATC∗ domain interacts with client proteins (24, 25). Since the Δ*nfuA* mutant of *E. coli* and *Pseudomonas aeruginosa* are each sensitive to oxidative stress and iron limitation conditions, NfuA was proposed to assist ErpA under severely unfavorable conditions (26).

Multiple lines of evidence suggest that the widely conserved CGFS-type (class II or monothiol) glutaredoxin, GrxD (Grx4) in *E. coli* also participates in Fe-S cluster biogenesis (Fig. 1*A*) (27, 28, 29, 30). In *Saccharomyces cerevisiae*, where much of the early work on CGFS-type Grxs was carried out, the homodimer of mitochondrial Grx5 (the yeast homolog of GrxD) accepts a [2Fe-2S] cluster from the scaffold protein Isu1 (the yeast homolog of IscU) (28). Grx5 interacts *in vitro* with the mitochondrial ATC proteins, Isa1 and Isa2, to transfer the cluster (29). Both *E. coli* and *Azotobacter vinelandii* GrxDs also accept [2Fe-2S] clusters from IscU scaffold proteins and deliver them to ATC or target Fe-S proteins *in vitro* (27, 30, 31, 32). *E. coli* GrxD can complement the phenotypes of a yeast Δ*grx5* mutant through heterologous expression, indicating evolutionary conservation of function (33). Additionally, abundant *in vivo* and *in vitro* studies have demonstrated that CGFS-type Grx proteins bind directly to BolA family members to form [2Fe-2S]-bridged heterodimers (27, 34). BolA and the related homolog IbaG can provide Cys and/or His ligands for cluster coordination within the heterodimer (27, 34). Deletion of *bolA* or *ibaG* in *E. coli* has only a minor effect on the Fe-S cluster dependent activity of respiratory complexes I and II (27, 35). Although deletion of *grxD* does not result in a growth defect under standard growth conditions, the Δ*grxD* mutation was reported to be synthetically lethal when combined with deletion mutations in the *isc* operon (36). Additionally, the Δ*grxD* strain is sensitive to iron depletion caused by the intracellular iron chelator 2,2′-bipyridyl (hereafter referred to as BiPy) (27). While these phenotypes point to a critical role for GrxD in Fe-S metabolism under stress conditions that disrupt Fe-S cluster homeostasis, whether and how GrxD integrates *in vivo* with the *E. coli* Fe-S cluster trafficking network is unknown.

**Figure 1:**
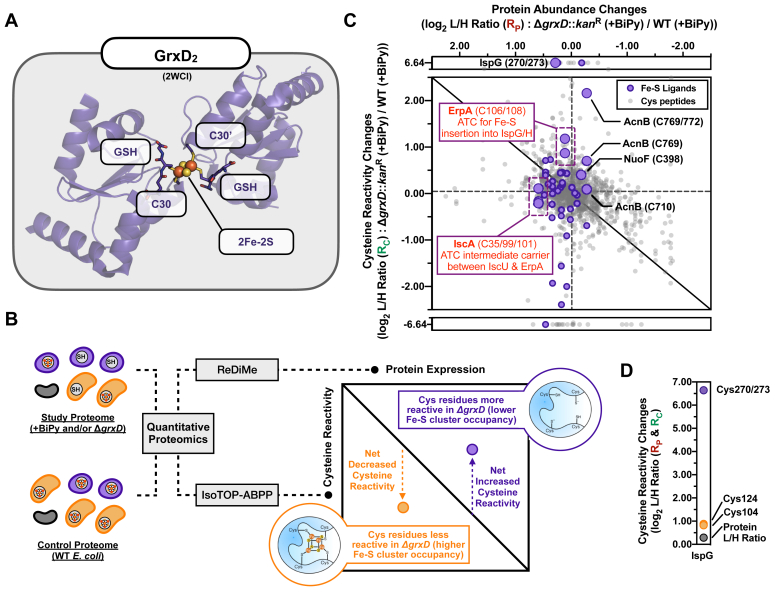
**Chemoproteomic analysis of the *Escherichia coli* Fe-S cluster-binding proteome in a*grxD*-deletion strain under BiPy stress.***A*, structure of the [2Fe-2S] cluster bound GrxD homodimer (PDB: 2WCI), showing the cluster ligated by a cysteine residue (C30, C30′) from each monomer and by thiols from two molecules of GSH. *B*, diagram showing how proteomic changes in protein abundance, determined by isotopic reductive dimethylation (ReDiMe), and changes in cysteine reactivity, determined by isoTOP-ABPP, are correlated on a two-dimensional plot to allow for the visualization of net changes in cysteine reactivity. For the Δ*grxD* strain, Fe-S cluster-binding cysteine residues with net increases in reactivity indicate sites with decreased Fe-S cluster occupancy, while those with net decreases in reactivity indicate sites with potentially increased Fe-S cluster occupancy. *C*, two-dimensional proteomic dataset of net cysteine reactivity changes across the *E. coli* proteome in the Δ*grxD* strain under 250 μM BiPy treatment compared to WT MG1655 strain under the same conditions. All quantified cysteine residues are plotted as *light gray circles*, while high-confidence cysteines that are known Fe-S cluster ligands are shaded in *purple*. Each data point represents the average cysteine reactivity change (*y-*axis, log_2_) from six replicates *versus* the average protein abundance change (*x-*axis, log_2_) from four replicates. Cysteine residues from ErpA and IscA proteins are highlighted within *purple dashed boxes*. *D*, IspG cysteine reactivity changes (average of six replicates) for the Fe-S cysteine ligand-containing peptide (Cys270/273, *purple*) and the nonligating cysteine residue–containing peptides (Cys124 and Cys 104, *orange*). The IspG protein L/H ratio (average of four replicates) is also indicated (*gray*) for reference. BiPy, 2,2′-bipyridyl; Fe-S, iron-sulfur; GrxD, glutaredoxin D; isoTOP-ABPP, isotopic tandem orthoganol proteolysis-activity-based protein profiling.

In this manuscript, we set out to understand the biological mechanism underlying the requirement for GrxD under iron limitation conditions as well as gain an understanding of how this unique iron starvation phenotype intersects with the known functions of GrxD in Fe-S trafficking. To achieve this we developed a multidisciplinary approach that relies on well-established genetic and biochemical techniques for studying Fe-S cluster metabolism coupled with cutting-edge chemoproteomic mass spectrometry (MS) analysis of the Fe-S proteome that provides global *in vivo* characterization of the function of GrxD in Fe-S cluster biogenesis under both normal and iron starvation conditions. Our studies revealed that (1) GrxD is required for maturation of only a specific subset of Fe-S proteins under iron depleted conditions, (2) GrxD is required for cluster delivery to ErpA under iron limitation, (3) GrxD is functionally distinct from other Fe-S trafficking proteins, and (4) GrxD Fe-S cluster binding is responsive to iron limitation. These findings lead us to conclude that GrxD provides a novel adaptation of the *E. coli* Fe-S delivery network in conditions that endanger Fe-S proteins. Our hypothesis is that under iron limitation conditions, *de novo* assembly of Fe-S clusters is lowered and that GrxD is there, as a reserve Fe-S pool, to ensure ErpA cluster loading. Thus, *in vivo* GrxD is crucial to ensure robustness of the Fe-S delivery network by maintaining the ErpA-dependent Fe-S cluster trafficking pathway.

## Results

### Deletion of *E. coli grxD* under BiPy stress impedes maturation of essential Fe-S client proteins

**Figure 2:**
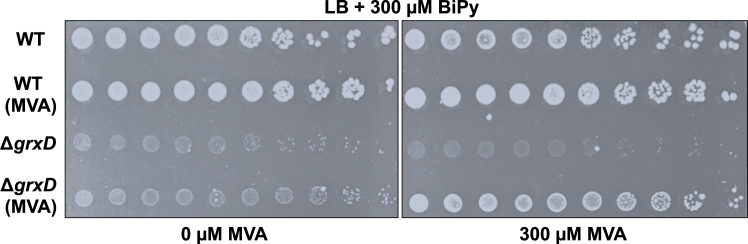
**Disrupted isoprenoid biosynthesis contributes to BiPy sensitivity in the *grxD*-deletion strain.** Serial dilutions of *Escherichia coli* Δ*grxD* and WT MG1655 strains that contain the heterologous MVA system for non-Fe-S–dependent isoprenoid synthesis, as well as parental control strains lacking the MVA system, were grown under iron starvation conditions on LB media containing 300 μM BiPy. Agar plates in the *right panel* also contained 300 μM mevalonate (MVA) to provide the necessary precursor for isoprenoid biosynthesis by the heterologous MVA system, while those in the *left panel* lacked mevalonate (see main text for additional details). BiPy, 2,2′-bipyridyl; GrxD, glutaredoxin D.

As previously described, we observed that the Δ*grxD* mutant is hypersensitive to the cell-permeable iron chelator BiPy, which is known to disrupt Fe-S cluster biogenesis (Fig. 2) (27). To understand the relationship between iron homeostasis and Fe-S cluster metabolism mediated by GrxD, we set out to fully analyze the Fe-S proteome in the BiPy-treated Δ*grxD* mutant. Loss of Fe-S cluster from protein binding sites frees the ligands formerly coordinated to the iron ions. In the case of cysteine residues, cluster loss results in a measureable increase in the reactivity of the Fe-S liganding cysteines toward thiol-modifying compounds. Therefore, we used a chemoproteomic strategy that quantifies the inherent differences in the *in vivo* reactivity of Fe-S cluster cysteine ligands in apo- *versus* holo-proteins (37). This approach enables proteome-wide monitoring of Fe-S cluster occupancy under a variety of experimental conditions (Figs. 1*B*, S1 and S2). This cysteine profiling workflow relies on two complementary and parallel chemoproteomic MS methods: (1) whole-proteome quantification using reductive dimethylation (ReDiMe), collected for four replicates, to monitor protein abundance changes between two experimental conditions (such as two different strains or two different growth conditions) (Fig. S1) and (2) isotopic tandem orthoganol proteolysis-activity-based protein profiling (isoTOP-ABPP), collected for six replicates, which uses a cysteine-reactive iodoacetamide-alkyne probe to monitor cysteine reactivity changes between the same two conditions (Fig. S2) (38, 39, 40). In our previous studies that validated this methodology, many *E. coli* Fe-S proteins displayed a decrease in abundance when Fe-S cluster biogenesis was impaired by mutations in the *isc* operon or iron depletion of the media (37). Protein abundance changes can occur as a result of transcriptional reprogramming and/or due to the instability of the apo-protein. Therefore, the cysteine reactivity changes that are measured by isoTOP-ABPP are corrected for protein abundance changes measured by ReDiMe to provide the net reactivity change for a given cysteine. Cysteine residues whose net reactivity is unchanged will track linearly with protein abundance along the diagonal correlation line in the two-dimensional plot (Fig. 1*B*). If Fe-S cluster binding by a protein is disrupted, the cysteine residues that act as ligands for the cluster will display net increases in reactivity and will lie above the correlation line (Fig. 1*B*). Using this approach, our previous studies confirmed that cysteine residues that are involved in Fe-S cluster ligation show net changes in reactivity, while bystander, nonligand cysteines in the same polypeptide tracked linearly with protein abundance changes (37). Therefore, the distance above or below the diagonal, which reflects the magnitude of the net reactivity change, is a useful proxy for Fe-S cluster binding regardless of changes in protein abundance (37).

Our initial experiments here confirmed that transient 2 h exposure of exponential phase liquid cultures to 250 μM BiPy perturbs the Fe-S proteome in the WT strain in a similar manner to that observed previously when cells were grown in iron depleted media (Fig. S3, *A*–*C*, Supplemental dataset S1, SI appendix, SI discussion) (37). In both BiPy-treated cells and in cells grown in iron depleted media, many Fur-regulated iron starvation response proteins were similarly induced, including significant induction of the SufA ATC and the rest of the Suf system under both conditions (Fig. S3, *D* and *E*) (37). Interestingly, we observe that in the WT strain grown in the presence of BiPy for 2 h, total GrxD protein abundance decreases by ∼30%, and GrxD cluster occupancy decreases moderately, as measured by a net increase in the reactivity of the GrxD cluster-ligating residue Cys30 (Fig. S3*F*).

Next, we compared wild-type and Δ*grxD* strains after transient 2 h exposure to 250 μM BiPy in exponential phase of growth (Figs. 1*C* and S4, Supplemental dataset S2). We observed that several important Fe-S proteins involved in oxidative respiration and metabolism, such as the [4Fe-4S] NuoF Fe-S subunit of complex I and the [4Fe-4S] enzyme aconitase B (AcnB), displayed lower Fe-S cluster occupancy (net increased cysteine reactivity) in the Δ*grxD* mutant (Figs. 1*C*, and S4*C*). Finally, we observed a large decrease in cluster binding (increased cysteine reactivity) for the isoprenoid biosynthetic enzyme, IspG that contains a [4Fe-4S] cluster (Fig. 1, *C* and *D*) (41). The Fe-S cluster cysteine ligands Cys270 and Cys273 of IspG both showed a large decrease in cluster binding, while the nonligand cysteines Cys104 and Cys124 did not show significant net cysteine reactivity changes (Fig. 1*D*).

Our proteomic data led us to reason that the BiPy-dependent growth defect of the Δ*grxD* mutant under iron starvation may be due to decreased IspG Fe-S cluster loading, thereby disrupting isoprenoid biosynthesis. To test this hypothesis, we sought to bypass any defect in the IspG activity through the introduction of a heterologous isoprenoid biosynthesis pathway containing genes for the yeast 5-diphospho-mevalonate decarboxylase, human 5-phosphomevalonate kinase, and yeast mevalonate (MVA) kinase into the chromosome of the Δ*grxD* mutant strain (42). The gene products of this synthetic operon do not require Fe-S clusters for activity, allowing instead for the synthesis of isoprenoids from exogenous MVA added to the growth media. This synthetic construct was previously shown to complement the lethality of an *E. coli* Δ*erpA* strain by restoring isoprenoid biosynthesis (20). We found that expression of the MVA pathway in the presence of exogenous MVA significantly improved Δ*grxD* growth under BiPy stress (Fig. 2), indicating that decreased isoprenoid biosynthesis contributes to BiPy sensitivity in the Δ*grxD* strain. Altogether, these results indicate that GrxD participates in maturation of a subset of client Fe-S proteins which include key/essential Fe-S client proteins.

### GrxD can provide Fe-S clusters to ErpA

**Figure 3:**
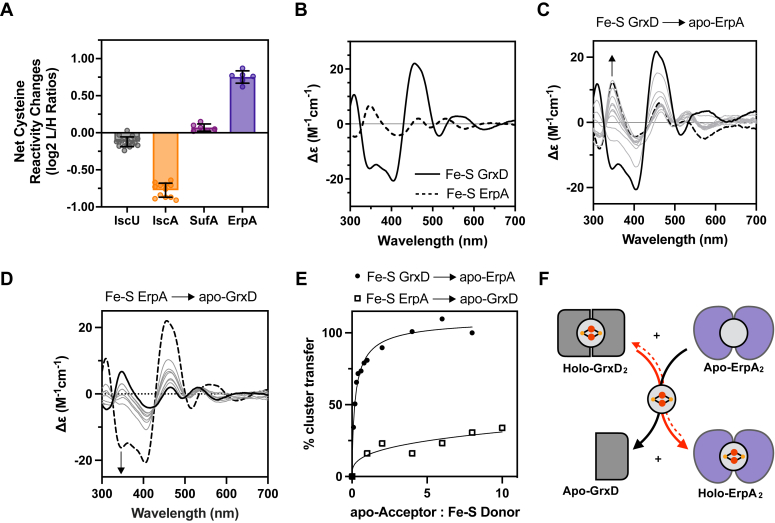
**GrxD can transfer Fe-S clusters to mature ErpA *in vitro*.***A*, *bar graph* of net cysteine reactivity changes for the high-confidence, fully labeled Fe-S cluster cysteine ligand–containing peptides of the Fe-S IscU scaffold (Cys37, Cys63, and Cys106, *gray*) and the ATC proteins IscA (Cys99/101 and Cys35, *orange*), SufA (Cys114/116, *magenta*), and ErpA (Cys106/108, *purple*) in the *Escherichia coli* Δ*grxD* strain under 250 μM BiPy treatment conditions. The average net cysteine reactivity changes (*bar height*) ± SD from six replicates is shown (*filled circles*). *B*, UV-visible CD spectra of 50 μM each of Fe-S GrxD (*solid line*) or Fe-S ErpA (*dashed line*), prepared separately and shown for comparison. *C*, 50 μM Fe-S GrxD (*solid black line*) was incubated with increasing amounts of apo-ErpA up to 8-fold excess (*gray lines*). Fifty micromolars of Fe-S ErpA was prepared separately and shown for reference (*dashed black line*). *Arrows* indicate direction of CD spectra changes at 346 nm as the acceptor/donor ratio is increased. *D*, 50 μM Fe-S ErpA (*solid black line*) was incubated with increasing amounts of apo-GrxD up to 8-fold excess (*gray lines*). Fifty micromolars of Fe-S GrxD was prepared separately and shown for reference (*dashed black line*). *Arrows* indicate direction of CD spectra changes at 346 nm as the acceptor/donor ratio is increased. *E*, percent cluster transfer from Fe-S GrxD to apo-ErpA (*solid circles*) or from Fe-S ErpA to apo-GrxD (*open squares*) as a function of the molar ratio of apo-acceptor to holo-donor. Percent cluster transfer was calculated from changes in CD spectra at 346 nm for each reaction. Separately prepared Fe-S holo-acceptor was used as a reference for 100%. *F*, scheme depicting unidirectional Fe-S cluster transfer from holo-GrxD_2_ to apo-ErpA_2_. *Solid arrows* indicate favored reactions, while *dashed lines* indicate unfavored reactions. *Red lines* indicate the flow of Fe-S clusters between holo and apo proteins. ATC, A-type Fe-S cluster carrier; BiPy, 2,2′-bipyridyl; Fe-S, iron-sulfur; GrxD, glutaredoxin D.

Our global chemoproteomic analysis of the Δ*grxD* mutant upon BiPy exposure revealed interesting changes not only in final Fe-S target proteins, but also in the Fe-S proteins of the Fe-S cluster trafficking network. In the Δ*grxD* mutant strain under BiPy stress, Fe-S cluster binding by the ErpA ATC was decreased (increased cysteine reactivity), while cluster binding by the IscA ATC was increased (decreased cysteine reactivity) (Figs. 1*C* and 3*A*). In contrast, the other Fe-S biogenesis factors such as, the the IscU scaffold and the SufA ATC, showed no change in cluster binding (*i.e.*, unchanged cysteine reactivity) in the Δ*grxD* mutant upon BiPy exposure (Fig. 3*A*). This result suggests GrxD is directly or indirectly required to maintain Fe-S cluster binding by ErpA under iron starvation stress.

To test if direct Fe-S cluster transfer from GrxD to ErpA can occur, we measured cluster transfer between purified GrxD and ErpA *in vitro* (Fig. 3, *B*–*F*). After anaerobic reconstitution with Fe-S cluster, each holo-protein donor was mixed with increasing amounts of the apo-protein acceptor under strictly anaerobic conditions. Any cluster transfer reactions between GrxD and ErpA were monitored anaerobically after reaching equilibrium by recording the UV-visible CD spectra in the range from 300 to 700 nm, where the Fe-S cluster-dependent CD features are specific for each Fe-S protein due to the interaction of the achiral Fe-S cluster with the chiral protein (Fig. 3*B*) (43). This methodology has been extensively used to analyze physiologically relevant Fe-S cluster formation and transfer reactions at multiple points along the trafficking pathway without separating the proteins (31, 43, 44, 45, 46, 47). Based on their individual spectra, we determined that purified Fe-S GrxD exhibits features distinct from purified Fe-S ErpA, thereby allowing us to track cluster occupancy in each protein during the cluster transfer reactions by monitoring changes in CD spectrum of each mixture (Fig. 3*B*).

We found that Fe-S GrxD efficiently transfers its cluster to apo-ErpA *in vitro* (Fig. 3*C*), achieving greater than 80% transfer of the GrxD Fe-S cluster to apo-ErpA upon addition of one equivalent of apo-ErpA (Fig. 3*E*). The reverse Fe-S cluster transfer reaction showed that transfer from Fe-S ErpA to apo-GrxD can occur but could not reach more than 40% of cluster transfer even at ten equivalents of apo-GrxD (Fig. 3, *D* and *E*). These results indicate Fe-S cluster transfer was more thermodynamically favorable from GrxD to ErpA than the transfer from ErpA to GrxD (Fig. 3*F*). We then tested if GrxD can transfer Fe-S clusters to another ATC protein. We performed the same *in vitro* cluster transfer assays using purified GrxD and purified SufA (Fig. S5). We found that Fe-S GrxD could transfer cluster to apo-SufA but the reaction required greater than a 5-fold molar excess of apo-SufA to reach completion, suggesting that Fe-S GrxD cluster transfer to apo-SufA is much less efficient than transfer from GrxD to ErpA (Fig. S5*B*). When we attempted to transfer cluster from Fe-S SufA to apo-GrxD, no cluster transfer was observed at any molar ratio tested (Fig. S5*C*). These results are consistent with our Fe-S proteome analysis and *in vivo* data suggesting that under BiPy stress, GrxD participates in the maturation of ErpA while maturation of SufA is not affected by GrxD (Fig. 3*A*).

### GrxD function cannot be replaced by the Fe-S scaffolds or other Fe-S cluster trafficking proteins

**Figure 4:**
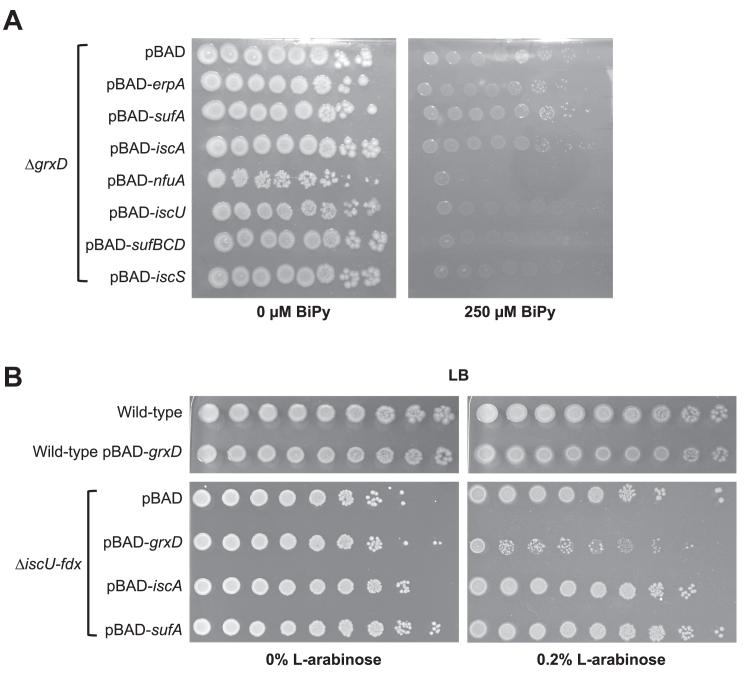
**GrxD function cannot be compensated by an alternative Fe-S scaffold or trafficking protein.***A*, the *Escherichia coli* Δ*grxD* mutant strain carrying pBAD plasmids expressing ATCs, NfuA, IscU, SufBCD, or the IscS cysteine desulfurase were grown overnight in LB. Culture densities were normalized to the same starting absorbance (turbidity), followed by serial dilution and spotting on LB agar plates (*left*) or LB agar supplemented with 250 μM BiPy (*right*). All agar plates also contained 0.2% L-arabinose to induce expression from pBAD plasmids. Plates were incubated at 37 °C for 18 h and were photographed. *B*, the WT MG1655 or the Δ*iscU-hscBA-fdx* strain transformed with various pBAD plasmids expressing GrxD, IscA, or SufA were grown overnight in LB. Culture densities were normalized to the same starting absorbance (turbidity), followed by serial dilution and spotting on LB agar plates (*left*) or LB agar plates supplemented with 0.2% L-arabinose (*right*). Plates were incubated at 30 °C for 20 h for the WT strain or for 44 h for the Δ*iscU-hscBA-fdx* strain in order to allow for similar final cell densities and were photographed. ATC, A-type Fe-S cluster carrier; BiPy, 2,2′-bipyridyl; Fdx, ferredoxin; Fe-S, iron-sulfur; GrxD, glutaredoxin D; LB, Lennox broth.

We next tested if ErpA or the other ATCs can act to bypass the requirement for GrxD function under BiPy stress (*i.e.*, act as multicopy suppressors of the Δ*grxD* BiPy sensitivity when overexpressed from a plasmid vector). We found that overexpression of ErpA, IscA, or SufA ATCs did not suppress the Δ*grxD* BiPy sensitivity to the same degree as providing GrxD in trans (Figs. 4*A* and S4*D*). Furthermore, overexpression of the NfuA Fe-S cluster trafficking protein, the Fe-S scaffolds IscU or SufBCD, or the IscS cysteine desulfurase did not suppress the Δ*grxD* BiPy sensitivity (Fig. 4*A*). Thus, it appears that GrxD cannot be replaced by other Fe-S cluster biogenesis components.

Next, we investigated whether GrxD can act as a multicopy suppressor of the Δ*iscUA-hscBA-fdx* mutant, which exhibits a slow growth phenotype even on rich Lennox broth (LB) media. Far from suppressing the Δ*iscUA-hscBA-fdx* slow growth phenotype, overexpressing GrxD in this mutant strain actually caused a significant impairment of growth compared to GrxD overexpression in the WT control strain (Fig. 4*B*). In contrast, overexpression of the ATC-II proteins IscA or SufA had no effect on the growth of the Δ*iscUA-hscBA-fdx* strain (Fig. 4*B*). Together, the multicopy suppressor results in Δ*grxD* and Δ*iscUA-hscBA-fdx* mutant strains point to a role for GrxD in Fe-S cluster metabolism that is distinct from the scaffolds or other Fe-S trafficking proteins.

### Δ*grxD* phenotypes diverge from WT, Δ*iscU*, and Δ*iscA* strains under normal growth conditions

**Figure 5:**
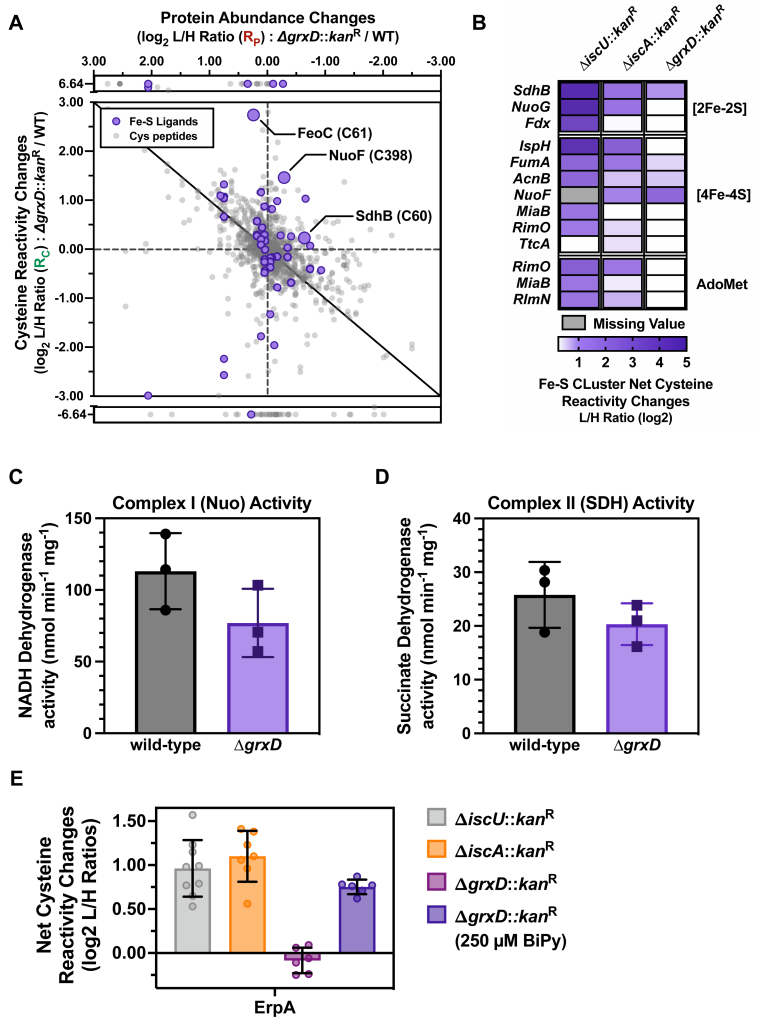
**The *Escherichia coli* Δ*grxD* mutant presents distinct phenotypes from those of the Δ*iscU* and Δ*iscA*.***A*, two-dimensional proteomic dataset of net cysteine reactivity changes across the *E. coli* proteome in the Δ*grxD* strain grown in LB compared to the WT MG1655 strain grown in the same conditions. All quantified cysteine residues are plotted as *light gray circles*, while high-confidence cysteines that are known Fe-S ligands are shaded in *purple*. Each data point represents the average cysteine reactivity change (*y*-axis, log_2_) from six replicates *versus* the average protein abundance change (*x*-axis, log_2_) from four replicates. *B*, heat map of net cysteine reactivity changes for a selection of annotated Fe-S clusters. Each cell represents the average of all quantified high-confidence, fully labeled Fe-S cluster cysteine ligand–containing peptides (average of six replicates) associated with that cluster. Increases in net cysteine reactivity in the indicated deletion strain are shaded in *purple*. Δ*iscU* and Δ*iscA* data from (37). *C*, NADH oxidase activity assayed from the WT and Δ*grxD* strains. The experiments were run in triplicate, and the S.E. values are shown (error bars). *D*, succinate dehydrogenase activity assayed from the WT and Δ*grxD* strains. The experiments were run in triplicate, and the S.E. values are shown (error bars). *E*, *bar graph* of net cysteine reactivity changes for the high-confidence, fully labeled Fe-S cluster cysteine ligand–containing peptides (Cys106/108) of the ATC protein ErpA in the Δ*iscU* (*gray*), Δ*iscA* (*orange*), and Δ*grxD* (*magenta*) mutant strains under standard conditions, and the Δ*grxD* mutant under 250 μM BiPy treatment conditions (*purple*). The average net cysteine reactivity changes (*bar height*) ± SD from six replicates is shown (*filled circles*). Δ*iscU* and Δ*iscA* data are from (37). ATC, A-type Fe-S cluster carrier; BiPy, 2,2′-bipyridyl; GrxD, glutaredoxin D; Fe-S, iron-sulfur; LB, Lennox broth.

To test if GrxD has any role in Fe-S cluster metabolism during growth under iron-replete conditions, we conducted chemoproteomic analysis of the Δ*grxD* and WT strains grown in rich LB media without addition of BiPy (Figs. 5*A* and S6*A*, Supplemental dataset S3). Net cysteine reactivity changes in the Δ*grxD* strain under normal growth conditions were limited compared to the WT strain (Figs. 5*A* and S6*B*). This result contrasts with the large global changes in cysteine reactivity previously observed in the Δ*iscU* mutant or even with the Δ*iscA* mutant strain that exhibits intermediate global changes (Fig. 5*B*) (37). A small number of specific Fe-S proteins did show net increases in cysteine ligand reactivity (decreased cluster binding) in the Δ*grxD* strain, including the [4Fe-4S] NuoF Fe-S subunit of complex I and the [2Fe-2S] SdhB Fe-S subunit of complex II (Fig. 5, *A* and *B*), which agrees with previous respiratory enzyme activity measurements in the Δ*grxD* strain as well as our Fe-S proteome results in the Δ*grxD* strain under BiPy stress (35). Consistent with this result, we also observed a mild decrease in complex I and complex II activity for the Δ*grxD* strain used in this study (Fig. 5, *C* and *D*). We also observed decreased cluster binding in FeoC, a [4Fe-4S] cluster-binding protein involved in regulating ferrous iron uptake (Fig. 5*A*). ErpA cluster binding was not significantly affected in the Δ*grxD* strain under normal conditions, in contrast with the Δ*iscU* and Δ*iscA* mutant strains where ErpA cluster binding was reduced (increased net cysteine reactivity) (Fig. 5*E*) (37). Interestingly, the magnitude of cluster loss for ErpA in the Δ*iscU* and Δ*iscA* strains under normal conditions is comparable to ErpA cluster loss in the Δ*grxD* strain under BiPy stress (Fig. 5*E*). This result suggests that IscU and IscA are primarily responsible for ErpA cluster delivery under normal conditions, while GrxD participates in ErpA cluster delivery exclusively under iron-starvation (BiPy) conditions. Together, these proteomic analyses indicate that deletion of *E. coli grxD* results in only very subtle perturbations to the Fe-S proteome under standard growth conditions, in contrast to *iscU* or *iscA* deletion.

**Figure 6:**
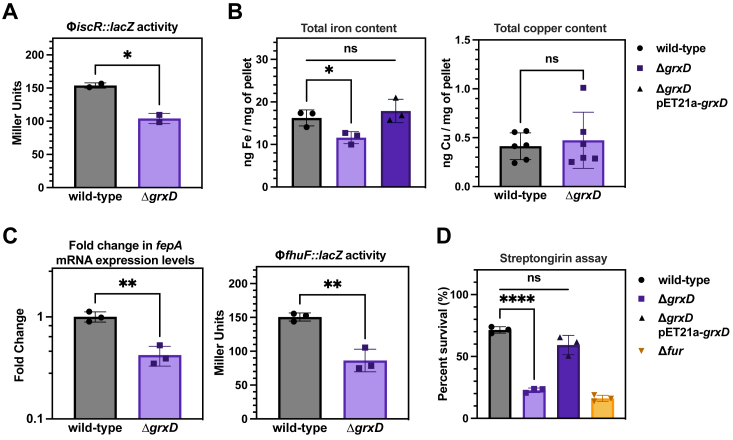
**Deletion of *grxD* disrupts iron homeostasis.***A*, β-galactosidase activity (expressed in Miller units) was measured in exponential phase for WT (*gray*) or Δ*grxD* mutant (*purple*) cells containing the *iscR* promoter-*lacZ* fusion. *p* value for Student’s *t* test was (∗) = 0.0148. Three biological replicates were performed for each experimental condition. *B*, total iron and copper content were measured in exponential phase cells of the indicated strains by graphite furnace AAS. Units shown are for dry weight of cell pellets collected. *p* value for Student’s *t* test was (∗) = 0.0273. *C*, the mRNA levels of *fepA* in exponential phase cells were measured by quantitative PCR. *p* value for Student's *t* test was (∗∗) = 0.0024. β-galactosidase activity (expressed in Miller units) of the *fhuF*::*lacZ* promoter-*lacZ* fusion was measured in exponential phase cells. *p* value for Student’s *t* test was (∗∗) = 0.0033. *D*, sensitivity to the iron-activated antibiotic streptonigrin was measured in the indicated strains. *p* value for Student’s *t* test was (∗∗∗∗) = <0.0001. AAS, atomic absorption spectroscopy; GrxD, glutaredoxin D.

As part of the chemoproteomic analysis, we also monitored total protein abundance changes across the two proteomes and found that 91 and 88 proteins displayed >2-fold increases or decreases in abundance, respectively, out of more than 1500 quantified proteins in the Δ*grxD* strain (Fig. S6*A*)*.* Gene ontology (GO) enrichment analysis demonstrated that many of the subset of proteins with >2-fold change were not involved in Fe-S–dependent pathways (Fig. S6*C*, Tables S1–S3). This is in contrast to the previous chemoproteomic data obtained in the Δ*iscU* and Δ*iscA* mutant strains where Fe-S–dependent pathways, such as thiamine biosynthesis, were specifically dysregulated. Deletion of *iscU* results in induction of both the *isc* and *suf* operons in a process mediated by the Fe-S cluster-binding transcriptional regulator, IscR (18, 37). Since no *suf* induction was observed in the Δ*grxD* strain under normal conditions, it suggests that under standard growth conditions, deletion of *grxD* does not induce the IscR regulon. To validate this observation, we also monitored transcriptional regulation of the *iscR* promoter, which is autoregulated by its own gene product, IscR. Expression of P_*iscR*_ is repressed by Fe-S cluster binding to the IscR protein, allowing IscR to directly respond to changes in the Fe-S cluster status of the cell (48, 49). We found that P_*iscR*_ promoter expression was slightly reduced by deletion of *grxD*, suggesting no impairment of cluster delivery to IscR and perhaps a slight enhancement of holo-IscR (leading to increased repression of the promoter) (Fig. 6*A*).

Proteomic analysis of the Δ*grxD* strain under normal growth conditions showed significantly decreased levels of several proteins (GpmA and SodA) that are repressed by Fur when labile iron is increased (Fig. S6*D*) (50, 51, 52). Additionally, FtnA protein, which is induced in a Fur-dependent manner when labile iron increases, was significantly elevated in the Δ*grxD* strain (Fig. S6*D*) (53). These changes in levels of iron homeostasis proteins in the Δ*grxD* strain are opposite of what is observed in the Δ*iscU* or Δ*iscA* strains, where multiple proteins in the Fur regulon are induced and FtnA expression is decreased (Fig. S6*D*).

Together these results indicate that *grxD* deletion does not result in a global increase in Fe-S cluster demand under normal conditions, in contrast to what is observed in the Δ*iscU* strain. However, cellular iron homeostasis may be impacted, as suggested by dysregulation of multiple proteins in the Fur regulon.

### Deletion of *E. coli grxD* increases the intracellular labile iron pool

We then further analyzed the impact of *grxD* deletion on cellular iron levels and iron homeostasis. Total cellular iron content was significantly reduced in the Δ*grxD* mutant compared the WT strain (Fig. 6*B*). Normal cellular iron content can be restored in the Δ*grxD* strain by addition of the *grxD* gene on a plasmid (Fig. 6*B*). In contrast, there was no significant difference in the levels of copper between the Δ*grxD* mutant and the WT strain (Fig. 6*B*). In *E. coli*, total cellular iron accumulation is dependent on iron uptake by numerous iron transport systems, such as those encoded by the *fep* and *fhu* loci, which are responsive to the iron-dependent transcriptional regulator, Fur (50, 51, 54, 55). Repression of these iron import pathways occurs under conditions of increased labile iron, which is the subpopulation of total iron that is directly sensed by Fur. Binding of iron to Fur increases its ability to bind to DNA and repress expression at target promoters. To confirm dysregulation of the Fur regulon and/or increased intracellular labile iron pools in the Δ*grxD* strain, we monitored the transcriptional expression of the Fur-regulated *fepA* and *fhuF* promoters using quantitative PCR (qPCR) for *fepA* and a chromosomally integrated *fhuF*-*lacZ* transcriptional fusion. The activity of both promoters was observed to be significantly repressed in the Δ*grxD* strain compared to the WT control (Fig. 6*C*). The increased labile iron pool in the Δ*grxD* strain was orthogonally confirmed with the iron-activated antibiotic streptonigrin. Increased labile iron in the cytosol (such as occurs in a Δ*fur* strain) will sensitize cells to killing by streptonigrin (56, 57). We found that the Δ*grxD* strain is highly sensitive to streptonigrin compared to the WT strain and was as sensitive as the Δ*fur* strain used as a positive control (Fig. 6*D*). Sensitivity to streptonigrin can be complemented by addition of the *grxD* gene on a plasmid (Fig. 6*D*). Taken together, these results demonstrate that the labile iron pool is elevated in the Δ*grxD* strain.

## Discussion

Here, we present multiple lines of evidence that GrxD supports Fe-S cluster delivery to ErpA under iron limitation conditions, and that this function allows *E. coli* to sustain growth under these conditions. First, under BiPy iron starvation stress, chemoproteomic analysis indicated that deletion of *grxD* decreases *in vivo* cluster binding by ErpA, but not IscA or SufA. While thiol oxidation events could impair iodoacetamide-alkyne labeling, resulting in false-negative identification of IscA or SufA cluster binding changes, we have previously shown that oxidation-induced changes at Fe-S cluster-binding sites occurring postlysis are minimal (37). On the other hand, oxidation events occurring before cell lysis, due to cellular thiol redox state changes as a result of the deletion of *grxD*, cannot be fully dismissed. Regardless, *in vitro*, we showed efficient unidirectional transfer of a [2Fe-2S] cluster from GrxD to ErpA. In contrast, SufA is not as efficiently loaded by GrxD *in vitro*, supporting the previous chemoproteomic assessment of minimal changes in either cluster binding or thiol oxidation for this ATC. Second, ErpA is involved in maturation of multiple Fe-S proteins, including AcnB, the Fe-S subunits of complexes I and II, and the IspG and/or IspH [4Fe-4S] isoprenoid biosynthesis enzymes, required for early steps in that pathway. Our Fe-S proteome analysis shows reduced Fe-S cluster loading of both AcnB and NuoF (complex I) under iron starvation stress in the Δ*grxD* mutant. Additionally, we directly observe that IspG cluster loading is decreased in the Δ*grxD* strain under iron starvation stress, suggesting cluster delivery from ErpA to this enzyme is compromised. Third, isoprenoid biosynthesis is a fundamental process in *E. coli* and IspG and IspH are essential Fe-S proteins, whose deletion compromises cell growth. By bypassing IspG and IspH function through the expression of a Fe-S–independent pathway for isoprenoid biosynthesis, the growth defect of the Δ*grxD* strain under iron starvation stress was largely suppressed. We conclude that under conditions of stress, such as iron-limitation, GrxD directly supports the essential cluster trafficking activities of ErpA by delivering clusters to ErpA, but not the other ATC proteins.

Previous phylogenetic, *in vivo*, and *in vitro* studies on ATCs provide insight into the preferential link between GrxD and ErpA (rather than with IscA or SufA). ATC proteins are classified in two different families, ATC-I and ATC-II. ErpA and other ATC-I proteins show preferential interactions with downstream apo-target proteins. In contrast, IscA, SufA, and other ATC-II proteins are principally connected to trafficking from their respective scaffold proteins (IscU or SufB). Therefore, GrxD can be viewed as an asset to secure the Fe-S cluster supply to ErpA *via* cluster transfer, especially under stress conditions that endanger Fe-S cluster biogenesis by the Isc system. This hypothesis is consistent with a recent report showing the *P*. *aeruginosa* Δ*grxD* strain is sensitive to some organic hydroperoxides and the redox cycling compound paraquat, which can all disrupt Fe-S metabolism (58). Together these results suggest a stress-response Fe-S cluster trafficking role for GrxD in *E. coli*.

Thus, like NfuA, GrxD would be a novel exemple of how Fe-S cluster delivery can be augmented by the addition of an accessory component acting on ErpA. However, while previous work has established that there is some level of functional redundancy between NfuA and the other ATC Fe-S cluster trafficking proteins, here we see a surprisingly different situation since the ATC and NfuA proteins failed to suppress the sensitivity of the Δ*grxD* mutant to BiPy iron starvation stress. This led us to question the role of GrxD as a simple carrier and instead propose that *in vivo*, GrxD might perform a distinct role in Fe-S metabolism as compared to the ATCs and NfuA. One hypothesis is that GrxD acts as an Fe-S cluster storage system that switches to a cluster carrier function under stress in order to mobilize the stored Fe-S clusters. While the exact line distinguishing Fe-S carrier from Fe-S storage protein would be difficult to draw, we can view GrxD as an extension that would refine the current trafficking model rather than as a replacement of the core network.

Our Fe-S proteome analyses demonstrate that GrxD protein levels decrease by 30% under iron limitation conditions, which may be at least partially explained by RhyB-mediated degradation of *grxD* mRNA under low iron conditions (59). Depletion of the *grxD* message would eventually lead to a decrease in protein levels, depending on the half-life of apo- and holo-GrxD. The slow depletion of the Fe-S cluster storage pool would help shift the dynamic equilibrium toward Fe-S cluster trafficking to the essential ErpA and other client proteins. The GrxD pool may provide a buffer to give the alternate Suf biogenesis system the opportunity to become fully operational. Once iron availability is restored, targeted degradation of *grxD* mRNA would stop due to Fur repression of RhyB and the GrxD Fe-S storage pool would be replenished. A similar regulatory circuit is observed for the iron storage protein FtnA, where its transcription is diminished in a Fur-dependent manner under low iron stress in order to facilitate iron release into the labile iron pool but then increased as the labile iron pool levels increases (53).

Here, we showed that cellular iron homeostasis, and specifically the labile iron pool, is disrupted by the Δ*grxD* mutation even under normal growth conditions. We hypothesize that, despite the decrease in total cellular iron content, the labile pool remains expanded due to the loss of GrxD. *E. coli* GrxD is the most abundant of the Fe-S proteins under normal growth conditions (60). Expansion of the labile iron pool in the Δ*grxD* mutant may suggest that GrxD contains a significant fraction of cellular iron under nonstressed growth conditions in LB. Loss of the highly abundant GrxD Fe-S binding protein could remove a key reservoir of Fe-S cluster binding capacity, leading to an increased labile iron pool. Future studies will aim at deciphering the exact mechanism for how the loss of GrxD may directly or indirectly lead to such a change in the labile iron pool. The elevated labile iron pool likely explains the observed decrease in total cellular iron because it leads to an inappropriate increase in transcriptional repression of iron uptake genes by Fe-Fur. Although the labile iron pool is expanded in the Δ*grxD* mutant, this does not help the mutant strain cope with iron starvation, indicating that incorporation of iron as an Fe-S cluster in GrxD is key to its role in the iron starvation response.

Regarding the question of how GrxD acquires its Fe-S cluster *in vivo* (*i.e.*, how is the storage pool maintained), ample *in vitro* evidence and *in vivo* studies from eukaryotes suggests that monothiol glutaredoxins interact with U-type scaffold proteins (like IscU) to obtain their cluster. However, in *E. coli*, deletion of the IscU scaffold does not decrease *in vivo* cluster binding by GrxD (37). In contrast, GrxD cluster occupancy does decrease under BiPy stress, possibly due to transfer of its Fe-S cluster to ErpA or other targets (Fig. S3*F*). Since *E. coli* also contains the SufBC_2_D scaffold, it is possible GrxD can access this alternate stress-responsive system in the absence of IscU (such as in the Δ*iscU* strain). Acquisition by GrxD of the Fe-S cluster from SufBC_2_D may also contribute to the hypersensitivity of the Δ*iscUA-hscBA-fdx* strain when GrxD is overexpressed (Fig. 4*B*). SufBC_2_D is the only remaining Fe-S biogenesis scaffold in the Δ*iscUA-hscBA-fdx* mutants. GrxD overexpression may sequester Fe-S clusters produced by SufBC_2_D and prevent them from efficiently trafficking to SufA or other downstream targets. This effect would block Suf function to create a situation analogous to an Δ*isc* Δ*suf* double mutation, which is lethal. All of these hypothetical models for preassembled cluster acquisition by GrxD are compatible with its possible role in Fe-S cluster storage. Future studies will focus on testing our Fe-S storage pool hypothesis, including discerning a mechanism for how GrxD may switch from storage to delivery. Lastly, these results provide new insight into monothiol Grx function *in vivo* that may prove useful for understanding the pathology of mitochondrial diseases such as variant nonketotic hyperglycinemia or congenital sideroblastic anemia caused by mutations in the human GLRX5 homolog of GrxD.

## Experimental procedures

### Construction of strains and plasmids and protein expression and purification

Detailed information on bacterial strain and plasmid construction as well as protein expression and purification are described in SI Experimental procedures.

### Chemoproteomics and MS

IsoTOP-ABPP cysteine reactivity and ReDiMe protein abundance chemoproteomic sample preparation and MS analysis of control, BiPy-treated, and Δ*grxD* mutant *E. coli* cultures were performed as described previously and in SI Experimental procedures (37). Cysteine reactivity data was collected as six replicates (two technical each of three biological replicates), while the protein abundance data was collected as four replicates (two technical each of two biological replicates).

### Growth medium and conditions

Bacterial strains were grown aerobically with shaking in LB and experiments were performed in biological triplicates. Serial dilution spot tests when overexpressing *grxD* were incubated at 30 °C to slow growth and allow for increased time point photographs. All other experiments were incubated at 37 °C. When required, kanamycin (50 μg/ml), ampicillin (100 μg/ml), or chloramphenicol (10 μg/ml) were added to the LB to select for antibiotic resistance cassettes. For cells grown under iron-limiting conditions, different concentrations of BiPy (Sigma) were added to the LB media from a concentrated stock made in ethanol. To provide the substrate needed for the non-Fe-S MVA pathway, exogenous Mevalonolactone (Sigma) dissolved in ethanol was added to the media at 300 μM final concentration. To induce expression from the pBAD plasmid, L-arabinose was added to the media at a final concentration of 0.2%. For serial dilution spot tests, overnight cultures were inoculated in LB medium with appropriate antibiotic selection and grown for 18 h. The *A*_600_ was measured and the cells were normalized to an *A*_600_ of 1.0 in LB media to serve as the 10^0^ dilution. From this normalized culture, 10 μl was transferred to 90 μl of fresh LB media. This process was repeated until a final serial dilution of 10^−9^ was reached for each strain. Five microliters from each dilution was spotted on an agar plate, dried, and then incubated at either 30 °C or 37 °C depending on the experiment. Incubation time was varied for different experiments depending on the strain being analyzed in order to allow for visualization of cell growth relative to control strains (see Figure legends for more details).

### Fe-S cluster reconstitution and cluster transfer assays

Protein (1 mM) to be reconstituted and 5 μM IscS were prereduced with 1 mM DTT for 1 h in an anaerobic chamber (Coy). An 8-fold molar excess of ferrous ammonium sulfate and 10-fold molar excess of L-cysteine were added to start the enzymatic reaction. Fe-S cluster reconstitution was monitored in a sealed anaerobic cuvette by UV-visible absorption and CD spectroscopies. Upon saturation of the [2Fe-2S] CD signal, GrxD was purified with a Q-sepharose anion exchange column while the ATCs were purified using a desalting column, under fully anaerobic conditions in both cases. Fractions containing Fe-S holo-protein were pooled and concentrated. Iron content was measured using the ferrozine assay (61). Typically, 90% of the GrxD_2_ or ATC dimer was reconstituted with [2Fe-2S] cluster under our conditions. For cluster transfer reactions, donor protein containing 50 μM [2Fe-2S] cluster was titrated with increasing molar ratios of apo-acceptor protein in a 300 μl total volume under strictly anaerobic conditions. Reactions were transferred under anaerobic conditions to a sealed 1 cm path length quartz cuvette and allowed to equilibrate for 15 min at room temperature before recording the CD spectrum of that molar ratio. Trial experiments indicated that all transfer reactions ceased changing (reached equilibrium) within 15 min. Titrations were performed on a Jasco J-815 spectrometer with a 290 to 700 nm range, 100 mdeg, 1 nm data pitch, continuous scanning mode, 200 nm/min scanning speed, 2 s response, and 10 nm bandwidth.

### Enzymatic assays

#### β-galactosidase assays

Three biological replicate cultures of *E. coli* were inoculated in LB medium with appropriate antibiotic selection and grown overnight. Overnight cultures were diluted 1:100 into fresh LB and incubated aerobically with shaking until an *A*_600_ of 0.2 to 0.35 was reached. At that point, β-galactosidase activity measurements were performed as previously described (62). Activity was calculated as Miller units.

#### NADH dehydrogenase activity

NADH dehydrogenase activity was adapted from a method described previously (63). Briefly, cells were harvested by centrifugation; resuspended in 50 mM phosphate buffer, pH 7.5; lysed using a French press; and frozen immediately in liquid nitrogen. NADH activity was assayed on the thawed samples by immediately adding d-NADH (200 mM) as substrate and by following *A*_340._

#### Succinate dehydrogenase activity

Cells were harvested by centrifugation; resuspended in 50 mM phosphate buffer, pH 7.5; and lysed using a French press. Following centrifugation (11,000 r.p.m. for 15 min at 4 °C), the supernatant was submitted to ultracentrifugation (45,000 r.p.m. for 2 h at 4 °C). Succinate dehydrogenase activity was assayed on the pellet fraction resuspended in 50 mM phosphate buffer, pH 7.5. Samples were preincubated for 30 min at 30 °C in 4 mM succinate, 1 mM KCN, 50 mM phosphate buffer, pH 7.5. The assay was performed by adding dichlorophenolindophenol (100 mM) and phenazine ethosulfate (1 mM) as substrate and by following *A*_600_.

### Total iron and copper quantification by graphite furnace atomic absorption spectroscopy

Biological triplicate overnight cultures were diluted 1:100 into 7 ml of fresh LB media and grown to an *A*_600_ of ∼0.2. The exponential phase cells were diluted 1:10 into 40 ml total volume in a sterile 250 ml baffled flask. These cultures were incubated aerobically for 2 h until *A*_600_ of 0.3 to 0.4. Then, 5 ml from each replicate were harvested by centrifugation at 4000*g* at 4 °C for 10 min, washed twice with 1 ml of PBS buffer pH 7.4 with 1 mM EDTA, and then twice more with PBS buffer pH 7.4 without EDTA. Pellets were weighed prior to being immediately frozen at −80 °C. Total iron and copper content were measured using a PinAAcle 900T AA Spectrometer (PerkinElmer). Standard concentrations for calibration curves were 0, 1, 2.5, 5, 7.5, 10 ppb and 0, 5, 10, 15, 20, 25 ppb for copper and iron, respectively. The cell pellets were thawed, resuspended in 0.2% nitric acid, and normalized to an absorbance of 1.0. An autosampler was used to dispense 20 μl of sample into the graphite furnace. If the sample was out of range of the standard curve, the autosampler automatically diluted it to obtain values within the calibration range.

### Steptonigrin assay

The streptonigrin sensitivity assay was done as described previously (64, 65). Briefly, biological triplicate cultures of *E. coli* were inoculated in LB medium with appropriate antibiotic selection and grown overnight. Overnight cultures were normalized to an *A*_600_ of 0.1 in 1 ml in either fresh LB containing 1 μg/ml streptonigrin from *Streptomyces flocculus* (Sigma) as a solution in dimethyl sulfoxide or in LB with an equivalent amount of dimethyl sulfoxide only (control cultures). Cultures were incubated aerobically with shaking for 18 h and then percent growth was measured by dividing the final *A*_600_ of the streptonigrin-treated cultures by the final *A*_600_ of the strain-matched control cultures.

### Total RNA isolation and qPCR analysis of *fepA* mRNA

Detailed information on RNA isolation and qPCR analysis are described in SI Experimental procedures.

## Data availability

The mass spectrometry proteomics data have been deposited to the ProteomeXchange Consortium (http://proteomecentral.proteomexchange.org) via the PRIDE partner repository (66) with the dataset identifier PXD053317. All other study data are included in the main text and Supporting information.

## Supporting information

This article contains supporting information (19, 20, 22, 37, 38, 39, 40, 42, 65, 67, 68, 69, 70, 71, 72, 73, 74, 75, 76, 77, 78).

## Conflict of interest

The authors declare that they have no conflicts of interest with the contents of this article.
