# Supplementary material for: Escherichia coli monothiol glutaredoxin GrxD replenishes Fe-S clusters to the essential ErpA A-type carrier under low iron stress
Source: J Biol Chem. 2024 Jun 27;300(8):107506. doi: 10.1016/j.jbc.2024.107506 (PMC11327457; doi:10.1016/j.jbc.2024.107506)
Supplement: Supporting Information [file mmc4.docx]

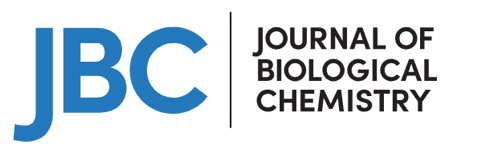


**Supporting Information for**

*Escherichia coli*monothiol glutaredoxin GrxD replenishes Fe-S clusters to the essential ErpA A-type carrier under low iron stress

Claire E. Fisher^1,#^, Daniel W. Bak^2,#^, Kennedy E. Miller^1^, Clorissa L. Washington-Hughes^1^, Anna M. Dickfoss^1^, Eranthie Weerapana^2^, Béatrice Py^3*^, and F. Wayne Outten^1,*^

^1^  University of South Carolina, Department of Chemistry and Biochemistry, Columbia, SC 29208 USA

^2^ Boston College, Department of Chemistry, Chestnut Hill, MA 02467 USA

^3^ Aix-Marseille Université-Centre National de la Recherche Scientifique (UMR7283), Laboratoire de Chimie Bactérienne, Institut de Microbiologie de la Méditerranée, Institut Microbiologie Bioénergies et Biotechnologie, 13009 Marseille, France.

^#^ These authors contributed equally

*Co-corresponding authors:

Béatrice Py, Email: [py@imm.cnrs.fr](mailto:py@imm.cnrs.fr)

F. Wayne Outten, Email: [woutten@sc.edu](mailto:woutten@sc.edu)

**This PDF file includes:**

SI Experimental Procedures

SI Discussion

SI Figures S1 to S6

SI Tables S1 to S5

SI References

**Other supporting materials for this manuscript include the following:**

Dataset S1 to S3

**SI Experimental Procedures**

**Construction of Strains and Plasmids**

The *E. coli* K-12 laboratory strain MG1655 and its derivatives were used in the present study (**Table S4**). Construction of deletion mutants was performed by λ RED recombination using gene-specific primers to amplify the chloramphenicol or kanamycin resistance cassettes from plasmids pKD3 and pKD4, respectively (**Tables S4, S5**) (67). The primers were designed such that the open reading frame of the target gene was deleted and replaced with the antibiotic resistance cassette. The PCR products were purified, concentrated, desalted, and transformed by electroporation into DY330 previously prepared using established protocols for induction of the λ RED system (68). Chromosomal integration of cassettes and deletion of the target gene were confirmed by colony PCR using primers flanking the mutation sites. Each mutation was moved from DY330 into MG1655 by P1 transduction. For monitoring labile intracellular iron pools, the Φ*fhuF*::*lacZ* construct was introduced into wild-type or ∆*grxD*::*cm*^R^ strains by P1 transduction (69). To provide a non-Fe-S pathway for isoprenoid biosynthesis, a heterologous construct containing genes for the yeast 5-diphospho-mevalonate decarboxylase, human 5-phosphomevalonate kinase, and yeast mevalonate kinase was moved by P1 transduction from DV1093 into the chromosome of the MG1655 derivatives used here (20,42).

For complementation studies, the plasmid pET21-*grxD* contains the region from -272 to +508 relative to the *grxD* transcriptional start site inserted into the *Bgl*II and *Xho*I sites of pET21a, was synthesized by Twist Biosciences. In this plasmid, the *grxD* locus is under the control of its native promoter and is not regulated by the inducible T7 promoter from the pET21 backbone. For over-expression of native *grxD* and *acnA*, the open reading frames were amplified from genomic DNA using primers GrxD_fwd and GrxD_rev or AcnA_fwd and AcnA_rev, respectively (**Table S5**). Both ORFs were cloned into the *Nco*I and *Hind*III restriction sties of pBAD/Myc-His C (Invitrogen). For construction of C-terminal His_6_-tagged GrxD for purification and biochemical experiments, the open reading frame was amplified from genomic DNA using primers GrxD_fwd and GrxD_rev_MycHis and cloned via HiFi Assembly (NEB) into the pBAD/Myc-His C plasmid (Invitrogen) at restriction sites *Nco*I and *Hind*III such that the C-terminus of GrxD is in frame with a Myc-His_6_ tag. ErpA was amplified from MG1655 chromosomal DNA as a template using ErpA_fwd and ErpA_rev primers (**Table S5**). SufA was amplified from MG1655 chromosomal DNA as a template using SufA_fwd and SufA_rev primers (**Table S5**). In both cases, purified PCR products were digested with *BamH*I and *Nde*I restriction enzymes and ligated into the corresponding sites of pET21a (Novagen) using T4 DNA ligase. The nucleotide sequence of the plasmid inserts was confirmed by DNA sequencing. Construction of pBAD-*iscA* and pBAD-*sufA* plasmids was described previously (19,22).

**Chemoproteomics and Mass Spectrometry**

*Generation of E. coli control, BiPy-treated, and ΔgrxD lysates for chemoproteomic analysis*: Biological triplicate cultures were inoculated in LB medium with appropriate antibiotic selection and incubated aerobically overnight. The cultures were diluted 1:100 into 40 mL total volume of fresh LB media in a sterile 250 mL baffled flask and incubated aerobically until an OD_600_ of 0.2 was reached. These exponential phase cells were diluted 1:5 into 160 mL total volume of fresh LB with or without 250 µM 2,2’-bipyridyl and incubated for 2 hours, typically reaching an OD_600_ of 0.3-0.4. In each case, a total OD_600_ of approximately 30 units was harvested. The cells were collected by centrifugation at 4000 x g at 4˚C for 15 minutes, washed in 1 mL of 1 x PBS pH 7.4, weighed, then immediately frozen at -80˚C until further analysis. Cell pellets were thawed then resuspended in 500 µL of PBS, lysed by sonication and centrifuged at 10,000 x g at 4˚C for 15 minutes to generate clarified lysates. Protein concentration was determined by Bradford assay (BioRad) and lysates were diluted to a concentration of 3 mg/mL with PBS pH 7.4 for isoTOP-ABPP analysis or 1 mg/mL for ReDiMe analysis.

*Preparation of ReDiMe labeled peptide samples for protein abundance analysis* (**Supplemental** **Fig. S1**): Samples for analysis of protein abundance were prepared in biological duplicate for each study condition. For each biological replicate, 100 µL (100 µg, 1 mg/mL) of both a control (w/ and w/o 250 µM BiPy) and study (250 µM BiPy and/or *grxD*) proteome were precipitated by the addition of 5 µL 100% trichloroacetic acid in water, vortexed and frozen (-80 ºC, overnight). After thawing, proteins were pelleted by centrifugation (15,000 g, 10 min, 4 ºC) and then the solvent was removed. Protein pellets were resuspended in 500 µL of ice-cold acetone by bath sonication and pelleted by centrifugation (5,000 x g, 10 min, 4 ºC). The solvent was removed and the pellet was allowed to air dry before being resuspended in 8 M Urea in 100 mM TEAB (30 µL). Reductive alkylation was performed by the sequential addition of 100 mM TEAB (70 µL) and 1.5 µL of 1 M DTT (65 ºC, 15 min), followed by 2.5 µL of 400 mM iodoacetamide (25 ºC, 30 min). Reactions were diluted with additional 100 mM TEAB (120 µL) and tryptic digestion performed with the addition of 2 µg of sequencing-grade trypsin (4 µL of 20 µg diluted in H_2_O) and 2.5 µL of 100 mM CaCl_2_ (37 ºC, overnight). After tryptic digest, reductive dimethylation was performed by the addition of 4 µL of 20% light (study proteome) or heavy (control proteome) formaldehyde and 20 µL of 0.6 M sodium cyanoborohydride (25 ºC, 2 hours) (40). The reaction was quenched by the addition of 8 µL ammonium hydroxide (25 ºC, 15 min). The light (study proteome) or heavy (control proteome) tryptic peptide samples were then combined, desalted on a Sep-Pak, and dried by speed-vac.

*Off-line high-pH ReDiMe-labeled peptide fractionation*: Samples were resuspended in 500 µL of high pH buffer A (95% H_2_O, 5% acetonitrile, 10 mM ammonium bicarbonate) and loaded onto a manual injection loop connected to an Agilent 1100 Series HPLC. Peptides were separated on a 25 cm Agilent Extend-C18 column using a 60 min gradient from 20-35% high pH buffer B (10% H_2_O, 90% acetonitrile, 10 mM ammonium bicarbonate) (70). Fractions were collected using a Gilson FC203B fraction collector into a 96 deep-well plate (0.6 min/well). Subsequent concatenation of every sixth well resulted in six pooled fractions that were dried by speed-vac and then resuspended in 50 µL of low pH buffer A (95% H_2_O, 5% acetonitrile, 0.1% formic acid).

*Isotopic IA-alkyne labeling, click chemistry, and enrichment of reactive cysteines* (**Supplemental Fig. S2)**: Samples for analysis of cysteine reactivity were prepared in biological triplicate for each study condition. For each biological replicate, 1 mL (2 mg/mL) of both a control and study (250 µM BiPy and/or ∆*grxD*) proteome were labeled with 100 μM (10 μL, 10 mM stock in DMSO) of either IA-alkyne light (IAL, study proteome) or IA-alkyne heavy (IAH, control proteome) for 1 h at room temperature (39). Probe labeled proteins were then conjugated a photo-cleavable biotin-azide tag (azo-tag) by Cu(I)-catalyzed [3 + 2] cycloaddition as previously reported (38,71). Briefly, 100 μM azo-tag (20 µL, 5 mM in DMSO), 1 mM TCEP (20 µL, 50 mM in water, freshly made), 100 μM TBTA (60 µL, 1.7 mM in 4:1 t-butanol:DMSO), and 2 mM CuSO_4_ (40 µL, 50 mM in water) were sequentially added to the labeled proteome. Reactions were vortexed and incubated at room temperature in the dark for 1 h with intermittent vortexing every 15 min. Light- and heavy-labeled proteomes were combined and centrifuged (6,500 *g*, 10 min, 4 °C) to collect a precipitated protein pellet. The supernatant was discarded and the protein pellet was resuspended in 500 μL of ice -cold methanol with sonication, followed by precipitation of the protein pellet by centrifugation (6,500 *g*, 10 min, 4 °C). This step was repeated, and the resulting washed pellet was redissolved in 1 mL of 1.2% w/v SDS in PBS by sonication followed by heating (5 min, 80-95 °C) to ensure complete solubilization. Samples were cooled to room temperature, diluted to 0.2 % w/v SDS with PBS (5 mL), and incubated with PBS-washed Streptavidin beads (200 μL of 50% aqueous slurry) overnight at 4 °C. Samples were allowed to warm to room temperature, pelleted by centrifugation (1,400 *g*, 3 min), and the supernatant discarded. Beads were then sequentially washed with 0.2% w/v SDS in PBS (5 mL x 1), PBS (5 mL x 3) and H2O (5 mL x 3) for a total of 7 washes.

*On-bead reductive alkylation, tryptic digestion, and UV cleavage of IA-labeled peptides*: Following the final wash, protein-bound streptavidin beads were resuspended in 6 M urea in PBS (500 μL) and reductively alkylated by sequential addition of 10 mM DTT (25 μL, 200 mM in H_2_O, 65 °C for 20 min) and 20 mM iodoacetamide (25 μL, 400 mM in H_2_O, 37 °C for 30 min) to each sample. Reactions were then diluted by addition of PBS (950 μL), pelleted by centrifugation (1,400 *g*, 3 min), and the supernatant discarded. Samples were subject to trypsin digestion by addition of 200 μL of a pre-mixed solution of 2 M urea in PBS, 1 mM CaCl_2_ (2 μL, 100 mM in H_2_O), and 2 μg of Promega Trypsin Gold (4 μL, 0.5 μg/μL in trypsin buffer). Samples were shaken overnight at 37 °C and then pelleted by centrifugation (1,400 *g*, 3 min) the next morning. Beads were then washed sequentially with PBS (500 μL x 3) and H_2_O (500 μL x 3). For photocleavage, the beads were resuspended in 250 µL of water and placed under UV lamp (360 nm) with gentle agitation via stirring for 2 hours. After UV cleavage, beads were centrifuged, and supernatant transferred to a new centrifuge tube. The beads were washed with water (75 μL, 2x), with the washes being combined with the previous supernatant, for a total eluted peptide sample volume of ~ 350 μL. Formic acid (17.5 μL) was added to a final concentration of 5% and samples were desalted on a Sep-Pak and dried by speed-vac before resuspension in 30 µL of low pH buffer A (95% H_2_O, 5% acetonitrile, 0.1% formic acid).

*LC-MS/MS of peptide samples*: Each biological sample was run in technical duplicate for both cysteine reactivity (6 replicates total) and protein abundance (4 replicates total) mass spectrometry proteomic analysis. LC-MS/MS was performed on an Orbitrap Exploris 240 mass spectrometer running Xcalibur v4.4 (Thermo Scientific) coupled to a Dionex Ultimate 3000 RSLCnano system. Each sample (5 µL) was injected directly onto an Acclaim PepMap 100 loading column. Peptides were eluted onto an Acclaim PepMap RSLC and separated with a 2-hour gradient from 5% to 25% of Buffer B (20% H_2_O, 80 % MeCN, 0.1% formic acid) in Buffer A (100% H_2_O, 0.1% formic acid) at a flow rate of 0.3 µL/min. The spray voltage was set to 2.1 kV. One full MS1 scan (120,000 resolution, 350-1800 m/z, RF lens 65%, AGC target 300%, automatic maximum injection time, profile mode) was obtained every 2 secs with dynamic exclusion (repeat count 2, duration 10 s), isotopic exclusion (assigned), and apex detection (30% desired apex window) enabled. A variable number of MS2 scans (15,000 resolution, AGC 75%, maximum injection time 100 ms, centroid mode) were obtained between each MS1 scan based on the highest precursor masses, filtered for monoisotopic peak determination, theoretical precursor isotopic envelope fit, intensity (5E4), and charge state (2-6). MS2 analysis consisted of the isolation of precursor ions (isolation window 2 m/z) followed by higher-energy collision dissociation (HCD, collision energy 30%). Each sample was subjected to a second injection and LC-MS/MS run in order to generate two technical replicates per biological sample.

*Database search and quantification of ReDiMe protein abundance peptide samples*: The tandem MS data was analyzed by the Thermo Proteome Discoverer V2.4 software package and searched using the SequestHT and Percolator algorithms against a UniprotKB database ([www.uniprot.org](http://www.uniprot.org)) of the *E. coli* K12 proteome (72,73). Trypsin was specified as the protease with a maximum of 2 missed cleavages. Peptide precursor mass tolerance was set to 10 ppm with a fragment mass tolerance of 0.02 Da. Oxidation of methionine (+15.995) as well as acetylation (+42.011) and/or methionine-loss (+131.040) of the protein N-terminus were set as dynamic modifications. Cysteine alkylation (+57.021) and either light (+28.031) or heavy (+34.063) dimethylation of lysine residues and peptide N-termini were set as static modifications for two independent sequest searches. The false discovery rate (FDR) for peptide identification was set to 1%. Protein light/heavy (L/H) ratios were assigned as the median value of all relevant peptide light/heavy ratios, calculated from the ratio of light and heavy dimethylated peptide precursor ion intensities. All protein abundance MS data was collected in four replicates (two technical replicates of two independent biological replicates).

*Database search and quantification of IA-labeled reactive cysteine peptide samples*: The tandem MS data was analyzed by the Thermo Proteome Discoverer V2.4 software package and searched using the SequestHT and Percolator algorithms against a UniprotKB database of *E. coli* K12 proteome (72,73). Trypsin was specified as the protease with a maximum of 2 missed cleavages. Peptide precursor mass tolerance was set to 10 ppm with a fragment mass tolerance of 0.02 Da. Oxidation of methionine (+15.995) as well as acetylation (+42.011) and/or methionine-loss (+131.040) of the protein N-terminus were set as dynamic modifications. Cysteine alkylation (+57.021) and either IAL (+285.159) or IAH (+291.179) modification with the photo-cleavable azo-tag were set as dynamic modifications for two independent sequest searches. The false discovery rate (FDR) for peptide identification was set to 1%. Peptide light/heavy (L/H) ratios were calculated from the ratio of IAL and IAH modified peptide precursor ion intensities. Annotation of cysteine function were generated from the Uniprot Protein Knowledgebase (UniProtKB) as described previously (74). All cysteine reactivity MS data was collected in six replicates (two technical replicates of three independent biological replicates).

*Statistical analysis and correlation of 2-dimensional proteomic data*: For both protein abundance (ReDiMe – 4 replicates) and cysteine reactivity (isoTOP-ABPP – 6 replicates) data, averaged L/H ratios are reported as log_2_ values (0 = no change in reactivity or abundance between the light and heavy sample). For cysteine reactivity data, peptides variants quantifying labeling of the same cysteine residue were averaged together (removing singleton values when another peptide contained a non-singleton ratio for a given replicate). Average log_2_ L/H ratios were generated by 1) manually removing singleton values from replicates when other non-singleton ratios are present in at least one other replicate, 2) correcting each replicate against its media value, excluding singletons, and 3) averaging the technical and biological replicates into a single average log_2_ L/H ratio. Confidence intervals were calculated for each log_2_ L/H ratio based on the coefficient of variance for each peptide or protein. High: C.o.V <0.5; Medium: 1.0 > C.o.V. > 0.5; Low: C.o.V. >1.0 (***see* Supplemental Datasets 1-3** for detailed description of confidence intervals). Averaged net cysteine reactivity data was calculated by subtracting the averaged protein abundance log_2_ L/H ratio (4 replicate values) from the corresponding averaged cysteine reactivity log_2_ L/H ratio (6 replicate values).

**Log_2_(Net Cys Reactivity L/H Ratio) = Log_2_(Cys Reactivity L/H Ratio) - Log_2_(Protein Abundance L/H Ratio)**

For bar graphs analysis of individual Fe-S cluster sites or cysteine ligands, each of the 6 replicate cysteine reactivity log_2_ L/H ratios from all relevant peptides were individually corrected by the average protein abundance log_2_ L/H ratio (4 replicate values).

*GO Analysis*: PANTHER (http://www.pantherdb.org) statistical overrepresentation was used to determine the biological processes that were most significantly overrepresented in an ID lists of those proteins upregulated (log_2_ L/H protein abundance greater than 1) for the Δ*grxD* (**Table S1**), Δ*iscU* (**Table S2**), and Δ*iscA* (**Table S3**) mutant strains compared to wild-type *E. coli* (75,76). Briefly, the number of expected protein IDs (Client Expect) from an input list of a given size (Client Input) is determine for all functional categories (PANTHER GO-Slim Biological Process), given the percentage of protein IDs for that category in the reference dataset (*E. coli* REFLIST). Overrepresentation (Client Fold Enrich) is given as the ratio of the observed over expected number of protein IDs for a functional category, such that a ratio above 1 indicates overrepresentation and a ratio below 1 indicates underrepresentation (Client over/under). A binomial test is used to determine the statistical significance of overrepresentation (Client P-value) for each functional category, with a P value cutoff of 0.05. This test makes no assumptions about how the datasets were generated and only assumes that the input and reference datasets were taken from the same protein population, or that each functional category should be equally well represented in both datasets.

**Protein expression and purification**

For ATCs, a single colony of BL21(DE3) freshly transformed with pET21a-SufA or pET21a_ErpA was grown overnight in LB with 100 µg/mL ampicillin. A 1:100 dilution of the overnight culture was used to inoculate sterile LB (1 L LB per 4 L baffled flask) with 100 μg/mL of ampicillin. Cultures were grown at 37°C with shaking until reaching an OD_600_ nm of 0.5 to 0.6, then expression was induced with 500 μM IPTG. The cultures were grown an additional 6 hours at 30°C with shaking before being harvested at 7,500 x g and 4°C for 10 minutes. Cell pellets from 1 L culture were collected, weighed, and stored in 50 mL conical tubes at -80°C. Cell pellets (from 1 L culture) were thawed at room temperature for 45 minutes and refrozen at -80°C for 15 minutes and this cycle was repeated three times. Then, cell pellets were resuspended in 5mL of Buffer A (25 mM Tris-HCl, 50 mM NaCl, 10 mM BME, pH 7.5) per gram of cell pellet. Resuspension was spun for 20 min at 31,000 x g at 4°C to remove cell debris. 2% streptomycin sulfate and 1 mM PMSF were added and freeze-thaw lysate was incubated on ice with occasional stirring for 20 minutes. The lysate was centrifuged at 31,000 x g for 40 minutes and the cleared lysate was loaded onto Q sepharose XL 16/10 column pre-equilibrated with Buffer A. Proteins were eluted with a linear gradient of salt up to 1M NaCl. SufA eluted between 0.4-0.5 M NaCl and ErpA eluted between 0.5-0.6M NaCl. Fractions containing SufA or ErpA were confirmed by SDS-PAGE, then pooled together. Pooled protein was concentrated using a 3,000 MWCO centrifugal device to an approximate concentration of 3mM. Concentrated A-type carrier was frozen drop-wise in 20 µl volumes in liquid nitrogen to form frozen beads. The beads were transferred to tube and stored at -80°C.

For GrxD, a single colony of Top10 freshly transformed with pBAD-*grxD*-Myc-His were grown overnight in LB with 100 µg/mL ampicillin. A 1:100 dilution of overnight culture was used to inoculate sterile LB to reach OD_600_ 0.5-0.6 and induced with 0.05% L-arabinose at 37°C for 6 hours and harvested at 7,500 x g for 10 minutes at 4°C. Pellets were stored in -80°C. The frozen pellets were resuspended in 5mL buffer (50 mM Tris-HCl, 500 mM NaCl, 20 mM imidazole, 10 mM BME, pH 7.4) per gram of pellet with 1 mM PMSF. Resuspension was sonicated with Branson Digital Sonifier Model 450 for 3 cycles of 10 s on, 30 s off for 1min at 50% amplitude. Resuspension was spun at 31,000 x g for 20 min at 4°C to remove cell debris. The His-tagged GrxD protein was purified with a GE HisTrap FF column with increasing concentration of elution buffer (50 mM Tris-HCl, 500 mM NaCl, 500 mM imidazole, 10 mM BME, pH 7.4) up to 100%. GrxD eluted between 0.3-0.4 M imidazole. Fractions were analyzed on SDS-PAGE. Fractions containing GrxD were pooled and concentrated before buffer exchanging into no imidazole buffer (50 mM Tris-HCl, 150 mM NaCl, 10 mM BME, pH 7.4). Protein was frozen in liquid nitrogen beads and stored in -80°C until further use. GrxD concentrations were determined using UV absorption at 280 nm. To remove residual cluster content in apo-proteins, the as-purified proteins were treated with a 50-fold molar ratio of EDTA and 20-fold molar ratio of potassium ferricyanide on ice for 60 minutes. Reaction was purified using a HiTrap desalting column and concentrated. Proteins were then pre-reduced with 20 mM DTT for 1 hour and buffer exchanged with 25 mM Tris-HCl, 150 mM NaCl, pH 7.8 buffer.

To purify IscS-His_6_, a single colony of BL21 (DE3) freshly transformed with pBH402 was used to inoculate LB media supplemented with 50 µg/ml kanamycin and incubated overnight. A 1:100 dilution from the overnight culture was used to inoculate two 4 L baffled flasks each containing 1 L of LB media with 50 µg/ml kanamycin. Cultures were incubated at 37˚C at 200 RPM until an OD_600_ of ~0.6 was reached. After addition of 100 µM IPTG (GoldBio) to induce protein expression and 100 µM pyridoxyl 5’-phosphate (PLP) (Sigma) to each flask, cultures were incubated at 25˚C for 20 hours at 200 RPM. Cells were harvested by centrifugation at 7,500 x g for 10 minutes at 4˚C. The cell pellets from each of the two 1 L flasks were combined in a sterile 50 ml falcon tube, weighed, then immediately frozen at -80˚C until purification. Frozen cell pellets were resuspended in 5 ml buffer A (50 mM Tris MES, 300 mM NaCl, 20 mM Imidazole, 5% Glycerol, 10 mM BME, pH 7.9) for every 1 g of cell pellet collected. 1 mM of PMSF was added into the resuspended cell pellet and let sit on ice for 30 minutes. The cells were lysed by sonication using the Branson Digital Sonifier Model 450 with 30 seconds on 60 seconds off 50% amplitude for 12 minutes total run time. After sonication, 10 µg/mL DNase I and 1 mM MgCl_2_ was added and followed by incubation on ice for 20 minutes. The cells were centrifuged at 31,000 x g for 40 minutes at 4˚C to pellet cell debris. The cleared lysate was loaded on a HisTrap 20 mL FF column (GE Healthcare). After washing with more Buffer A, IscS-His_6_ was eluted with increasing concentration of % buffer B (50 mM Tris MES, 300 mM NaCl, 500 mM Imidazole, 5% Glycerol, 10 mM BME, pH 7.9). IscS eluted off the column at an imidazole range of 0.13-0.25 M. Protein fractions containing IscS were analyzed using SDS-PAGE and fractions were pooled together and concentrated using a 30,000 MWCO centrifugal device. Imidazole was buffer exchanged away from the protein using 50 mM Tris MES, 300 mM NaCl, 5% Glycerol, 10 mM BME, pH 7.9. Protein concentration was determine using the Bradford assay. The protein at final concentration of 3.02 mM was flash frozen by pipetting 20 µL drops into liquid nitrogen. The protein was stored at -80˚C until use in Fe-S cluster reconstitution experiments.

**Total RNA Isolation from Bacteria**

Biological triplicate overnight cultures were diluted 1:100 into 5 mL LB in a sterile 50 mL conical tube and incubated aerobically until an OD_600_ of 0.2 was reached. These cultures were then diluted 1:10 into 30 mL total volume of LB media in a sterile 125 mL baffled flask and incubated aerobically for two hours until the cultures reached an OD_600_ of 0.4-0.5. Next, 20 mL from each replicate culture was centrifuged at 4000 x g at 4˚C for 10 minutes. RNA isolation was carried out based on a previously published protocol with minor adjustments (77). Briefly, cell pellets were resuspended in solution A (0.5% SDS, 20 mM sodium acetate, 10 mM EDTA, pH 5.5). Acid phenol pH 4.3 (Sigma) was added followed by incubation for 35 minutes at 65˚C. After centrifugation at 13,000 x g for 1 minute to separate phases, the upper phase was transferred to a new 1.5 mL Eppendorf tube. Trace phenol was removed by adding 500 µL of chloroform, followed by centrifugation, and transfer of the upper aqueous phase to a new 1.5 mL Eppendorf tube. 30 µL of 3M sodium acetate (pH 5.3) was added followed by 1 mL of chilled 100% ethanol to precipitate total RNA. After centrifugation at 13,000 x g at 4˚C for 5 minutes, the supernatant was removed, and the RNA pellet was washed with 70% ethanol. The RNA pellet was resuspended in 400 µL solution A followed by ethanol precipitation and this process was repeated twice more. RNA concentration and purity was quantified using a BioTek Synergy H1 Hybrid Plate Reader using the 260 nm and 260/280 ratio, respectively. To remove residual contaminating DNA, the TURBO DNA-*free* Kit (Invitrogen AM1907) was used to DNase treat 10 µg of total RNA following the manufacturers protocol. After treatment, RNA concentration and purity was remeasured. The DNase treated RNA samples were stored in the -80˚C for future use.

**qPCR Analysis of *fepA* mRNA**

qPCR was performed in the BioRad CFX Connect Real-Time System using the iTaq Universal SYBR Green One-Step Kit (BioRad 172-5151). The iTaq SYBR green mastermix, reverse transcriptase, DNase treated RNA, primers and nuclease-free H_2_O were added per manufacturers protocol to reach a final volume of 20 µL per reaction. 300 ng of total DNase treated RNA and 300 nM of each forward and reverse primer were used per reaction. Total RNA isolated from each biological replicate was analyzed separately by qPCR. The thermal cycling protocol was chosen per manufacturers recommended cycle for the BioRad CFX Connect using the SYBR scan mode. This program consisted of 10 minutes at 50˚C, 1 minute at 95˚C, followed by 40 cycles of 10 seconds at 95˚C, 30 seconds at 60˚C with a plate read. A melt curve analysis was performed to ensure primer specificity using a 0.5˚C gradient every 5 seconds with a plate read beginning at 65˚C and ending at 95˚C. ∆Cq analysis was performed to analyze change in expression levels of *fepA* against the reference gene (*hcaT*). Primer sequences for *fepA* and *hcaT* expression can be found in **Table S5**. ∆∆Cq analysis was performed to calculate fold change in expression levels between the different wild-type and mutant backgrounds as described in the figure legend.

**SI Discussion**

**Comparison of iron starvation created through iron depeleted media or by addition of the iron chelator BiPy.**

These experiments also allowed us to conclude that the cellular response was widely similar independent of the methods used to disrupt iron homeostasis. We observed that two hours of BiPy treatment largely mimics the proteomic effects of iron limitation (37). In terms of the Fe-S proteome, global net changes in cysteine reactivity were not significantly different between the two iron starvation stress conditions, as both showed elevated cysteine reactivity (reduced cluster binding) compared to the untreated control grown in iron-replete media (**Supplemental Fig. S3A-C**). BiPy incubation for two hours clearly increased cysteine reactivity in a number of Fe-S proteins, including multiple [4Fe-4S] enzymes like the TCA cycle enzyme FumA, the isoprenoid biosynthetic enzyme IspH, and the NuoG subunit of Complex I (**Supplemental Fig. S3A,B**). Interestingly, BiPy also was effective at increasing cysteine reactivity of the [2Fe-2S] proteins NuoG and SdhB, which were largely unaffected in the iron-depleted media condition. In contrast, mononuclear iron enzymes such as LuxS and Def had increased cysteine reactivity in iron-depleted media but not under BiPy exposure for two hours (**Supplemental Fig. S3B**). These subtle differences suggest that BiPy may directly chelate iron from Fe-S cluster biogenesis proteins or Fe-S cluster metalloenzymes such that both [2Fe-2S] and [4Fe-4S] clusters are affected but cannot effectively access iron used in mononuclear iron enzymes (at least during a two hour exposure). As expected, strains grown in BiPy or in iron-depleted media both showed increased abundance of proteins involved in iron uptake and iron starvation stress, including much of the Fur regulon and the *suf* operon (**Supplemental Fig. S3D,E**).

**SI References**

19. D. Vinella, C. Brochier-Armanet, L. Loiseau, E. Talla, F. Barras, Iron-sulfur (Fe/S) protein biogenesis: phylogenomic and genetic studies of A-type carriers. *PLoS Genet.* **5**, e1000497 (2009).

20. L. Loiseau, *et al.*, ErpA, an iron–sulfur (Fe–S) protein of the A-type essential for respiratory metabolism in *Escherichia coli*. *Proc. Natl. Acad. Sci. U. S. A.* **104**, 13626–13631 (2007).

22. S. Angelini, *et al.*, NfuA, a new factor required for maturing Fe/S proteins in *Escherichia coli* under oxidative stress and iron starvation conditions. *J. Biol. Chem.* **283**, 14084–14091 (2008).

37. D. W. Bak, E. Weerapana, Monitoring Fe-S cluster occupancy across the *E. coli* proteome using chemoproteomics. *Nat. Chem. Biol.* **19**, 356–366 (2023).

38. E. Weerapana, *et al.*, Quantitative reactivity profiling predicts functional cysteines in proteomes. *Nature* **468**, 790–795 (2010).

39. M. Abo, C. Li, E. Weerapana, Isotopically-Labeled Iodoacetamide-Alkyne Probes for Quantitative Cysteine-Reactivity Profiling. *Mol. Pharm.* **15**, 743–749 (2018).

40. P. J. Boersema, R. Raijmakers, S. Lemeer, S. Mohammed, A. J. R. Heck, Multiplex peptide stable isotope dimethyl labeling for quantitative proteomics. *Nat. Protoc.* **4**, 484–494 (2009).

42. N. Campos, *et al.*, *Escherichia coli* engineered to synthesize isopentenyl diphosphate and dimethylallyl diphosphate from mevalonate: a novel system for the genetic analysis of the 2-C-methyl-d-erythritol 4-phosphate pathway for isoprenoid biosynthesis. *Biochem. J.* **353**, 59–67 (2001).

65. Y. Wu, F. W. Outten, IscR Controls Iron-Dependent Biofilm Formation in *Escherichia coli* by Regulating Type I Fimbria Expression. *J. Bacteriol.* **191**, 1248–1257 (2009).

67. K. A. Datsenko, B. L. Wanner, One-step inactivation of chromosomal genes in *Escherichia coli* K-12 using PCR products. *Proc. Natl. Acad. Sci. U. S. A.* **97**, 6640–6645 (2000).

68. D. Yu, *et al.*, An efficient recombination system for chromosome engineering in *Escherichia coli*. *Proc. Natl. Acad. Sci. U. S. A.* **97**, 5978–5983 (2000).

69. E. Massé, F. E. Escorcia, S. Gottesman, Coupled degradation of a small regulatory RNA and its mRNA targets in *Escherichia coli*. *Genes Dev.* **17**, 2374–2383 (2003).

70. A. Edwards, W. Haas, Multiplexed Quantitative Proteomics for High-Throughput Comprehensive Proteome Comparisons of Human Cell Lines. *Methods Mol. Biol. Clifton NJ* **1394**, 1–13 (2016).

71. E. Weerapana, A. E. Speers, B. F. Cravatt, Tandem orthogonal proteolysis-activity-based protein profiling (TOP-ABPP)--a general method for mapping sites of probe modification in proteomes. *Nat. Protoc.* **2**, 1414–1425 (2007).

72. J. K. Eng, A. L. McCormack, J. R. Yates, An approach to correlate tandem mass spectral data of peptides with amino acid sequences in a protein database. *J. Am. Soc. Mass Spectrom.* **5**, 976–989 (1994).

73. L. Käll, J. D. Canterbury, J. Weston, W. S. Noble, M. J. MacCoss, Semi-supervised learning for peptide identification from shotgun proteomics datasets. *Nat. Methods* **4**, 923–925 (2007).

74. D. W. Bak, M. D. Pizzagalli, E. Weerapana, Identifying Functional Cysteine Residues in the Mitochondria. *ACS Chem. Biol.* **12**, 947–957 (2017).

75. H. Mi, S. Poudel, A. Muruganujan, J. T. Casagrande, P. D. Thomas, PANTHER version 10: expanded protein families and functions, and analysis tools. *Nucleic Acids Res.* **44**, D336-342 (2016).

76. H. Mi, A. Muruganujan, J. T. Casagrande, P. D. Thomas, Large-scale gene function analysis with the PANTHER classification system. *Nat. Protoc.* **8**, 1551–1566 (2013).

77. M. Kawano, T. Oshima, H. Kasai, H. Mori, Molecular characterization of long direct repeat (LDR) sequences expressing a stable mRNA encoding for a 35-amino-acid cell-killing peptide and a cis-encoded small antisense RNA in *Escherichia coli*. *Mol. Microbiol.* **45**, 333–349 (2002).

78. E. G. Mueller, P. M. Palenchar, C. J. Buck, The role of the cysteine residues of ThiI in the generation of 4-thiouridine in tRNA. *J. Biol. Chem.* **276**, 33588–33595 (2001).

**SI Figures**

**Supplemental Figure 1** **ReDiMe workflow for protein abundance analysis.** Labeling of tryptic peptides from two biological samples by reductive dimethylation, with either isotopically light (CH_2_O) or heavy (CH^13^D_2_O) formaldehyde, allows for quantitative determination of changes in protein abundance by LC-MS/MS analysis.

**Supplemental Figure 2** **IsoTOP-ABPP workflow for cysteine reactivity analysis.** Labeling of proteins from two biological samples with isotopically light (IA-Light) or heavy (IA-Heavy) iodoacetamide alkyne followed by CuACC with a photocleavable biotin-azide (PC-Biotin-Azide), allows for protein enrichment on streptavidin beads, on-bead trypsin digestion, peptide release by UV cleavage and quantitative determination of changes in cysteine reactivity by LC-MS/MS analysis.

**Supplemental Figure 3** **Protein abundance and net cysteine reactivity changes associated with 250 µM BiPy treatment for two hours in the *E. coli* wild-type MG1655 strain**. *A*, Two-dimensional proteomic dataset of net cysteine reactivity changes for the *E. coli* proteome in the wild-type strain MG1655 grown with 250 µM for two hours compared to 0 µM BiPy treatment. All quantified cysteine residues are plotted as light gray circles, while high-confidence known Fe-S ligands are shaded in purple. *B*, Heat map of net cysteine reactivity changes in the wild-type strain grown under 250 µM BiPy treatment for two hours or in Fe-depleted media for a selection of annotated Fe-S clusters. Each cell represents the average of all quantified high-confidence, fully-labeled Fe-S cluster cysteine ligand-containing peptides (avg. of 6 replicates) associated with that cluster. Clusters with increases in net cysteine reactivity in the Fe-depleted condition compared to iron-replete control are shaded in purple. Fe-depletion data from (37). *C*, Violin plot of global net changes in cysteine reactivity (avg. of 6 replicates) for all quantified Fe-S cluster ligands for the control and Fe-depletion (orange) and 250 µM BiPy (purple) treatments. The median R value is displayed as a dashed line in the violin plot. Significance is calculated as *** p < 0.005 (p = 9.49E-6), paired t-test (two-tailed), with an n of 21, 30, and 39 for the control., Fe-depletion, and 250 µM BiPy conditions, respectively.  Control and Fe-depletion data from (37). *D*, Protein abundance changes (avg. of 4 replicates) displayed as a waterfall plot of the log_2_ L/H ratios (R_p_) for two hour 250 µM treatment versus 0 µM BiPy treated *E. coli* wild-type strain. Upregulated (R_p_ > 1.0) and downregulated (R_p_ < -1.0) proteins are highlighted in orange and purple, respectively. *E*, Heat map of protein abundance changes (avg. of 4 replicates) for a selection of operons regulated by the iron-responsive and Fe-S-responsive transcription factors Fur and IscR, respectively. Each cell represents the average of all quantified protein log_2_ L/H ratios (R_p_) for that operon in the specified strain. Increased protein abundance in the deletion strain is shaded in orange and decreases shaded in purple. Control and Fe-depletion data from (37). *F,* Bar graph of net cysteine reactivity changes for the high-confidence, Fe-S cluster cysteine ligand-containing peptide of GrxD (Cys30) in the ∆*iscU* mutant strain under standard growth conditions (gray) or the wild-type MG1655 strain under 250 µM BiPy treatment conditions (purple). The average net cysteine reactivity changes (bar height) +/- SD from 6 replicates across one or more peptides involved in cluster ligation are shown (filled circles). ∆*iscU* data is from (37).

**Supplemental Figure 4** **Protein abundance and net cysteine reactivity changes associated with *grxD*-deletion after two hours of BiPy treatment**. *A*, Protein abundance changes (avg. of 4 replicates displayed as a waterfall plot of log_2_ L/H ratios (R_p_) for the *E. coli* Δ*grxD* strain versus the wild-type MG1655 strain both under 250 µM BiPy treatment. Upregulated (R_p_ > 1.0) and downregulated (R_p_ < -1.0) proteins are highlighted in orange and purple, respectively. *B*, Violin plot of global net changes in cysteine reactivity for all quantified Fe-S cluster ligands (avg. of 6 replicates) for the Δ*grxD* strain in the presence of 250 µM BiPy for two hours (orange). The median R value is displayed as a dashed line in the violin plot. *C*, Heat map of net cysteine reactivity changes for a selection of annotated Fe-S cluster proteins in the Δ*grxD* strain after 250 µM BiPy treatment for two hours. Each cell represents the average of all quantified high-confidence, fully-labeled Fe-S cluster cysteine ligand-containing peptides (avg. of 6 replicates) associated with that cluster. Increases in net cysteine reactivity in the Δ*grxD* deletion strain are shaded in purple. *D*, The *E. coli* wild-type MG1655 strain and ∆*grxD* mutant with or without the pBAD-*grxD* plasmid were grown overnight in LB. Culture densities were normalized to the same starting optical density (turbidity) followed by serial dilution and spotting on LB agar plates (left) or LB agar plates supplemented with 250 µM BiPy (right). Plates were incubated at 30°C for 18 hours and were photographed.

**Supplemental Figure 5** **Fe-S cluster transfer reactions between [2Fe-2S]-GrxD and SufA.** *A*, Titration of 50μM [2Fe-2S]-GrxD (solid black line) with increasing concentrations of apo-SufA up to 16:1 molar ratio. The spectrum of 50μM [2Fe-2S]-SufA (dashed black line) is shown for reference. Arrow indicates the direction of change as apo-SufA increases. *B*, The percent cluster transfer from [2Fe-2S]-GrxD to apo-ErpA as a function of the change in Δε at 346nm (filled circles) and from [2Fe-2S]-GrxD to apo-SufA as a function of the change in Δε at 345nm (open diamonds). Data for ErpA transfer is reproduced from Figure 3E. *C*, Titration of 50μM [2Fe-2S]-SufA (solid black line) with increasing concentrations of apo-GrxD up to 8:1 molar ratio. The spectrum of 50μM [2Fe-2S]-GrxD reconstituted separately (dashed black line) is shown for reference.

**Supplemental Figure 6** **Protein abundance and net cysteine reactivity changes associated with *grxD*-deletion under standard growth conditions**. *A*, Protein abundance changes (avg. of 4 replicates) displayed as a waterfall plot of the log_2_ L/H ratios (R_p_) for exponential phase *E. coli* Δ*grxD* versus wild-type MG1655 strains grown under standard conditions in LB. Upregulated (R_p_ > 1.0) and downregulated (R_p_ < -1.0) proteins are highlighted in orange and purple, respectively. *B*, Violin plot of global net changes in cysteine reactivity (avg. of 6 replicates) for all quantified Fe-S cluster ligands for the Δ*iscU* and Δ*iscA* (orange) and Δ*grxD* (purple) strains. The median R value is displayed as a dashed line in the violin plot. Significance is calculated as *** p < 0.005 (p = 8.79E-7), paired t-test (two-tailed), with an n of 30, 26, and 49 for Δ*iscU*, Δ*iscA*, and Δ*grxD* strains, respectively.  Δ*iscU* and Δ*iscA* data from (37). *C*, GO analysis of processes enriched in proteins upregulated in the Δ*iscU,* Δ*iscA,* and Δ*grxD* strains. Δ*iscU* and Δ*iscA* data are from (37). The apparent induction of the FNR and FlhDC regulons (processes marked by an asterisk) in the ∆*grxD* strain is due to hidden mutations that we identified during the course of this study by genome sequencing. The wild-type MG1655 strain used as a control for proteomic experiments contains a previously unidentified indel in the *fnr* gene that affect FNR function (**data not shown**). The ∆*grxD* strain has a wt copy of *fnr*, but has an IS1 mobile element insertion inside of the gene *dgcJ*, inactivating the gene and perturbating the FlhDC-regulated *fliD* gene (**data not shown**). All genetic and biochemical experiments in the main text were collected using the correct matching parent strain for the ∆*grxD* mutant as a control (that is the control and mutant strains are isogenic except for the ∆*grxD* mutation). *D*, Heat map of averaged protein abundance changes (increase – orange, decreased – purple) from 4 replicates for all proteins in a selection of operons regulated by the iron-responsive and Fe-S-responsive transcription factors Fur and IscR in the ∆*iscU*, ∆*iscA*, and ∆*grxD* mutant strains. ∆*iscU* and ∆*iscA* data are from (37).

**SI Tables**

**Table S1 GO analysis upregulated proteins (log2 L/H Ratio > 1) in the Δ*grxD* mutant**

| **GO biological process** | ***E. coli* - REFLIST (4392)** | **Client (98)** | **Client Expect** | **Client over/**  **under** | **Client Fold Enrich** | **Client**  **P-value** | **Client FDR** |
| --- | --- | --- | --- | --- | --- | --- | --- |
| aerotaxis (GO:0009454) | 4 | 4 | 0.09 | + | 44.82 | 1.40E-05 | 2.31E-03 |
| energy taxis (GO:0009453) | 4 | 4 | 0.09 | + | 44.82 | 1.40E-05 | 2.19E-03 |
| regulation of chemotaxis (GO:0050920) | 4 | 4 | 0.09 | + | 44.82 | 1.40E-05 | 2.09E-03 |
| positive regulation of post-translational protein modification (GO:1901875) | 3 | 3 | 0.07 | + | 44.82 | 1.92E-04 | 2.15E-02 |
| regulation of post-translational protein modification (GO:1901873) | 3 | 3 | 0.07 | + | 44.82 | 1.92E-04 | 2.08E-02 |
| taxis (GO:0042330) | 26 | 18 | 0.58 | + | 31.03 | 1.63E-19 | 1.28E-16 |
| chemotaxis (GO:0006935) | 26 | 18 | 0.58 | + | 31.03 | 1.63E-19 | 1.02E-16 |
| locomotion (GO:0040011) | 26 | 18 | 0.58 | + | 31.03 | 1.63E-19 | 8.54E-17 |
| archaeal or bacterial-type flagellum-dependent cell motility (GO:0097588) | 52 | 22 | 1.16 | + | 18.96 | 3.65E-20 | 5.71E-17 |
| cilium or flagellum-dependent cell motility (GO:0001539) | 52 | 22 | 1.16 | + | 18.96 | 3.65E-20 | 3.81E-17 |
| bacterial-type flagellum-dependent cell motility (GO:0071973) | 50 | 21 | 1.12 | + | 18.82 | 3.25E-19 | 1.46E-16 |
| regulation of protein modification process (GO:0031399) | 10 | 4 | 0.22 | + | 17.93 | 1.81E-04 | 2.10E-02 |
| nitrate assimilation (GO:0042128) | 20 | 8 | 0.45 | + | 17.93 | 8.38E-08 | 2.63E-05 |
| nitrate metabolic process (GO:0042126) | 20 | 8 | 0.45 | + | 17.93 | 8.38E-08 | 2.39E-05 |
| cell motility (GO:0048870) | 59 | 23 | 1.32 | + | 17.47 | 2.03E-20 | 6.36E-17 |
| regulation of response to external stimulus (GO:0032101) | 11 | 4 | 0.25 | + | 16.3 | 2.42E-04 | 2.53E-02 |
| nitrate assimilation (GO:0042128) | 20 | 8 | 0.45 | + | 17.93 | 8.38E-08 | 2.63E-05 |
| nitrate metabolic process (GO:0042126) | 20 | 8 | 0.45 | + | 17.93 | 8.38E-08 | 2.39E-05 |
| cell motility (GO:0048870) | 59 | 23 | 1.32 | + | 17.47 | 2.03E-20 | 6.36E-17 |
| regulation of response to external stimulus (GO:0032101) | 11 | 4 | 0.25 | + | 16.3 | 2.42E-04 | 2.53E-02 |
| nitrogen cycle metabolic process (GO:0071941) | 24 | 8 | 0.54 | + | 14.94 | 2.64E-07 | 6.89E-05 |
| reactive nitrogen species metabolic process (GO:2001057) | 24 | 8 | 0.54 | + | 14.94 | 2.64E-07 | 6.36E-05 |
| bacterial-type flagellum-dependent swarming motility (GO:0071978) | 23 | 7 | 0.51 | + | 13.64 | 2.57E-06 | 4.47E-04 |
| bacterial-type flagellum organization (GO:0044781) | 27 | 8 | 0.6 | + | 13.28 | 5.60E-07 | 1.10E-04 |
| bacterial-type flagellum assembly (GO:0044780) | 18 | 5 | 0.4 | + | 12.45 | 1.10E-04 | 1.33E-02 |
| regulation of locomotion (GO:0040012) | 25 | 6 | 0.56 | + | 10.76 | 4.39E-05 | 5.73E-03 |
| anaerobic respiration (GO:0009061) | 61 | 12 | 1.36 | + | 8.82 | 3.62E-08 | 1.26E-05 |
| response to external stimulus (GO:0009605) | 164 | 21 | 3.66 | + | 5.74 | 2.12E-10 | 8.32E-08 |
| cellular respiration (GO:0045333) | 114 | 14 | 2.54 | + | 5.5 | 4.91E-07 | 1.02E-04 |
| energy derivation by oxidation of organic compounds (GO:0015980) | 130 | 15 | 2.9 | + | 5.17 | 3.84E-07 | 8.59E-05 |
| signal transduction (GO:0007165) | 92 | 9 | 2.05 | + | 4.38 | 3.08E-04 | 3.12E-02 |
| signaling (GO:0023052) | 98 | 9 | 2.19 | + | 4.12 | 4.75E-04 | 4.65E-02 |
| generation of precursor metabolites and energy (GO:0006091) | 209 | 15 | 4.66 | + | 3.22 | 8.32E-05 | 1.04E-02 |
| response to chemical (GO:0042221) | 386 | 25 | 8.61 | + | 2.9 | 1.32E-06 | 2.44E-04 |
| biological_process (GO:0008150) | 3408 | 92 | 76.04 | + | 1.21 | 2.18E-05 | 3.10E-03 |
| Unclassified (UNCLASSIFIED) | 984 | 6 | 21.96 | - | 0.27 | 2.18E-05 | 2.97E-03 |

**Table S2 GO analysis of upregulated proteins (log2 L/H Ratio > 1)in the Δ*iscU* mutant (from SI Reference (37)).**

| **GO biological process** | ***E. coli* - REFLIST (4392)** | **Client**  **Iput**  **(63)** | **Client Expect** | **Client over/**  **under** | **Client Fold Enrich** | **Client**  **P-value** | **Client FDR** |
| --- | --- | --- | --- | --- | --- | --- | --- |
| thiamine diphosphate metabolic process (GO:0042357) | 11 | 5 | 0.16 | + | 31.69 | 1.87E-06 | 7.31E-04 |
| thiamine diphosphate biosynthetic process (GO:0009229) | 11 | 5 | 0.16 | + | 31.69 | 1.87E-06 | 6.50E-04 |
| thiamine biosynthetic process (GO:0009228) | 12 | 5 | 0.17 | + | 29.05 | 2.62E-06 | 8.20E-04 |
| thiamine-containing compound biosynthetic process (GO:0042724) | 13 | 5 | 0.19 | + | 26.81 | 3.58E-06 | 1.02E-03 |
| thiamine metabolic process (GO:0006772) | 15 | 5 | 0.22 | + | 23.24 | 6.34E-06 | 1.42E-03 |
| thiamine-containing compound metabolic process (GO:0042723) | 15 | 5 | 0.22 | + | 23.24 | 6.34E-06 | 1.32E-03 |
| primary alcohol biosynthetic process (GO:0034309) | 16 | 5 | 0.23 | + | 21.79 | 8.23E-06 | 1.36E-03 |
| iron-sulfur cluster assembly (GO:0016226) | 22 | 6 | 0.32 | + | 19.01 | 1.86E-06 | 9.69E-04 |
| metallo-sulfur cluster assembly (GO:0031163) | 22 | 6 | 0.32 | + | 19.01 | 1.86E-06 | 8.31E-04 |
| alcohol biosynthetic process (GO:0046165) | 19 | 5 | 0.27 | + | 18.35 | 1.66E-05 | 2.37E-03 |
| methionine biosynthetic process (GO:0009086) | 16 | 4 | 0.23 | + | 17.43 | 1.49E-04 | 1.50E-02 |
| methionine metabolic process (GO:0006555) | 16 | 4 | 0.23 | + | 17.43 | 1.49E-04 | 1.45E-02 |
| sulfur compound biosynthetic process (GO:0044272) | 66 | 11 | 0.95 | + | 11.62 | 5.93E-09 | 9.29E-06 |
| sulfur amino acid metabolic process (GO:0000096) | 34 | 5 | 0.49 | + | 10.25 | 1.91E-04 | 1.77E-02 |
| pyrimidine-containing compound biosynthetic process (GO:0072528) | 37 | 5 | 0.53 | + | 9.42 | 2.74E-04 | 2.09E-02 |
| sulfur compound metabolic process (GO:0006790) | 131 | 17 | 1.88 | + | 9.05 | 7.97E-12 | 2.50E-08 |
| organic hydroxy compound biosynthetic process (GO:1901617) | 39 | 5 | 0.56 | + | 8.94 | 3.42E-04 | 2.55E-02 |
| primary alcohol metabolic process (GO:0034308) | 47 | 6 | 0.67 | + | 8.9 | 8.58E-05 | 9.96E-03 |
| water-soluble vitamin biosynthetic process (GO:0042364) | 67 | 8 | 0.96 | + | 8.32 | 8.14E-06 | 1.42E-03 |
| vitamin biosynthetic process (GO:0009110) | 68 | 8 | 0.98 | + | 8.2 | 8.99E-06 | 1.41E-03 |
| alpha-amino acid catabolic process (GO:1901606) | 63 | 7 | 0.9 | + | 7.75 | 4.80E-05 | 6.01E-03 |
| water-soluble vitamin metabolic process (GO:0006767) | 80 | 8 | 1.15 | + | 6.97 | 2.68E-05 | 3.66E-03 |
| vitamin metabolic process (GO:0006766) | 81 | 8 | 1.16 | + | 6.89 | 2.92E-05 | 3.81E-03 |
| pyrimidine-containing compound metabolic process (GO:0072527) | 62 | 6 | 0.89 | + | 6.75 | 3.47E-04 | 2.53E-02 |
| aspartate family amino acid metabolic process (GO:0009066) | 64 | 6 | 0.92 | + | 6.54 | 4.07E-04 | 2.90E-02 |
| cellular amino acid catabolic process (GO:0009063) | 76 | 7 | 1.09 | + | 6.42 | 1.44E-04 | 1.50E-02 |
| alpha-amino acid biosynthetic process (GO:1901607) | 120 | 9 | 1.72 | + | 5.23 | 6.82E-05 | 8.22E-03 |
| alpha-amino acid metabolic process (GO:1901605) | 198 | 13 | 2.84 | + | 4.58 | 5.34E-06 | 1.29E-03 |
| cellular amino acid biosynthetic process (GO:0008652) | 144 | 9 | 2.07 | + | 4.36 | 2.54E-04 | 1.99E-02 |
| cellular amino acid metabolic process (GO:0006520) | 260 | 15 | 3.73 | + | 4.02 | 4.07E-06 | 1.06E-03 |
| carboxylic acid biosynthetic process (GO:0046394) | 208 | 11 | 2.98 | + | 3.69 | 2.02E-04 | 1.71E-02 |
| organic acid biosynthetic process (GO:0016053) | 209 | 11 | 3 | + | 3.67 | 2.10E-04 | 1.73E-02 |
| small molecule biosynthetic process (GO:0044283) | 311 | 16 | 4.46 | + | 3.59 | 7.49E-06 | 1.38E-03 |
| organonitrogen compound biosynthetic process (GO:1901566) | 522 | 24 | 7.49 | + | 3.21 | 1.22E-07 | 9.58E-05 |
| cellular component biogenesis (GO:0044085) | 316 | 14 | 4.53 | + | 3.09 | 1.51E-04 | 1.43E-02 |
| carboxylic acid metabolic process (GO:0019752) | 510 | 18 | 7.32 | + | 2.46 | 2.43E-04 | 1.95E-02 |
| organonitrogen compound metabolic process (GO:1901564) | 1003 | 35 | 14.39 | + | 2.43 | 2.71E-08 | 2.83E-05 |
| oxoacid metabolic process (GO:0043436) | 534 | 18 | 7.66 | + | 2.35 | 6.51E-04 | 4.53E-02 |
| organic acid metabolic process (GO:0006082) | 545 | 18 | 7.82 | + | 2.3 | 7.31E-04 | 4.98E-02 |
| organic substance biosynthetic process (GO:1901576) | 911 | 27 | 13.07 | + | 2.07 | 1.19E-04 | 1.33E-02 |
| small molecule metabolic process (GO:0044281) | 884 | 26 | 12.68 | + | 2.05 | 1.95E-04 | 1.74E-02 |
| cellular biosynthetic process (GO:0044249) | 889 | 26 | 12.75 | + | 2.04 | 2.01E-04 | 1.75E-02 |
| biosynthetic process (GO:0009058) | 926 | 27 | 13.28 | + | 2.03 | 1.32E-04 | 1.43E-02 |
| nitrogen compound metabolic process (GO:0006807) | 1547 | 40 | 22.19 | + | 1.8 | 7.27E-06 | 1.42E-03 |
| cellular metabolic process (GO:0044237) | 1972 | 48 | 28.29 | + | 1.7 | 6.52E-07 | 4.09E-04 |
| metabolic process (GO:0008152) | 2205 | 49 | 31.63 | + | 1.55 | 1.06E-05 | 1.57E-03 |

**Table S3 GO analysis of upregulated proteins (log2 L/H Ratio > 1) in the Δ*iscA* mutant (from SI Reference (37)).**

| **GO biological process** | ***E. coli* - REFLIST (4392)** | **Client Input (55)** | **Client Expect** | **Client over/**  **under** | **Client Fold Enrich** | **Client**  **P-value** | **Client FDR** |
| --- | --- | --- | --- | --- | --- | --- | --- |
| negative regulation of termination of DNA-templated transcription (GO:0060567) | 9 | 4 | 0.11 | + | 35.49 | 1.38E-05 | 8.64E-03 |
| negative regulation of protein-containing complex disassembly (GO:0043242) | 9 | 4 | 0.11 | + | 35.49 | 1.38E-05 | 7.20E-03 |
| iron-sulfur cluster assembly (GO:0016226) | 22 | 6 | 0.28 | + | 21.78 | 8.28E-07 | 2.59E-03 |
| metallo-sulfur cluster assembly (GO:0031163) | 22 | 6 | 0.28 | + | 21.78 | 8.28E-07 | 1.30E-03 |
| negative regulation of cellular component organization (GO:0051129) | 16 | 4 | 0.2 | + | 19.96 | 8.76E-05 | 2.75E-02 |
| sulfur compound metabolic process (GO:0006790) | 131 | 9 | 1.64 | + | 5.49 | 4.35E-05 | 1.70E-02 |
| response to abiotic stimulus (GO:0009628) | 254 | 12 | 3.18 | + | 3.77 | 7.08E-05 | 2.47E-02 |
| cellular component organization (GO:0016043) | 412 | 17 | 5.16 | + | 3.29 | 8.49E-06 | 8.87E-03 |
| cellular component organization or biogenesis (GO:0071840) | 469 | 18 | 5.87 | + | 3.06 | 1.13E-05 | 8.82E-03 |
| biological regulation (GO:0065007) | 770 | 23 | 9.64 | + | 2.39 | 2.55E-05 | 1.14E-02 |

| **Table S4 Strains and Plasmids** | | |
| --- | --- | --- |
| **Strain** | **Genotype** | **Source or SI Reference** |
| MG1655-1 (CF4) | *fnr* ^-^ | Laboratory strain |
| MG1655-2 (CF173) | *dgcJ ^-^* | Laboratory strain |
| CF8 | MG1655-2 ∆*grxD*::*kan*^R^ | This study |
| CF159 | MG165-2 ∆*grxD::kan*^R^ pET21a-*grxD* | This study |
| CF171 | MG1655-2 ∆*grxD*::*cm*^R^ | This study |
| CF169 | MG1655-2 ∆*fur* | (65) |
| BP129 | MG1655 MVA^+^ (*kan*^R^) | (20,42) |
| CF187 | MG1655-2 MVA^+^ (*kan*^R^) | This study |
| CF175 | MG1655-2 ∆*grxD::cm*^R^ MVA^+^ (kan^R^) | This study |
| CF184 | MG1655-2 Φ*fhuF*::*lacZ-kan^R^* | Provided by E. Masse (69) |
| CF185 | MG1655-2 Φ*fhuF*::*lacZ-kan^R^* ∆*grxD*::*cm*^R^ | This study |
| YW103 | MG1655-2 ∆*iscU-fdx::kan^R^* | This study |
| CF188 | MG1655-2 ∆*iscU-fdx::kan^R^* pBAD/Myc-His C |  |
| CF104 | MG1655-2 ∆*iscU-fdx::kan^R^* pBAD-*grxD* | This study |
| CF189 | MG1655 ∆*iscU-fdx::kan^R^*  pBAD-*iscA* | This study |
| CF190 | MG1655 ∆*iscU-fdx::kan^R^*  pBAD-*sufA* | This study |
| BL21 (DE3) | Protein expression strain | Novagen |
| KM9 | DH5α pBAD-*grxD/*Myc-His C | This study |
| KM10 | DH5α pET21-*erpA* | This study |
| KM11 | DH5α pET21-*sufA* | This study |
| **Plasmid** | **Details** | **Source or SI Reference** |
| pBAD-*grxD* | *grxD* cloned into pBAD vector under control of *ara* promoter | This study |
| pBAD-*grxD-*Myc-His | *grxD* cloned into pBAD vector in frame with a C-terminal Myc-His tag. Expression of *grxD*-Myc-His is under control of *ara* promoter. | This study |
| pBAD-*iscA* | *iscA* cloned into pBAD vector under control of *ara* promoter | (19,22) |
| pBAD-*sufA* | *sufA* cloned into pBAD vector under control of *ara* promoter | (19,22) |
| pBH402 | IscS-His_6_ expression vector | (78) |
| pET21a-*grxD* | Entire *grxD* locus cloned into pET21a vector such that *grxD* is under control of its native promoter. | This study |
| pET21a-*erpA* | *erpA* cloned into pET21a for protein overexpression | This study |
| pET21a-*sufA* | *sufA* cloned into pET21a for protein overexpression | This study |

| **Table S5 Oligionucleotides used in this study** | | |
| --- | --- | --- |
| **Name** | **Sequence (5’- 3’)** | **Description** |
| ErpA_fwd | TAAACATATGAGTGATGACGTAGCACTGCCGC | For cloning *erpA* into pET21a |
| ErpA_rev | ATAGGGATCCTTAGATACTAAAGGAAGAACCGCAA |  |
| fepA_qPCR_forward | CACCTGGTTCCGTAACGATTA | For qPCR analysis of the *fepA* gene |
| fepA_qPCR_reverse | GCACGTTATCCCACTGATAGAG |  |
| GrxD_fwd | ggctaacaggaggaattaacATGAGCACCACTATCGAAAAAATCC | For amplifying *grxD* from the genome. Uppercase nucleotides correspond to region that overlaps with *grxD* and lowercase nucleotides correspond to region that overlaps with the pBAD/Myc-His C plasmid. |
| GrxD_rev | atgagtttttgttctacgtaagcTTATTCCGCGTCCGGCTC |  |
| GrxD_rev_MycHis | atgagtttttgttctacgtaTTCCGCGTCCGGCTCTTC | For amplifying *grxD* from the genome. Uppercase nucleotides correspond to region that overlaps with *grxD* and lowercase nucleotides correspond to region that overlaps with the pBAD/Myc-His C plasmid. Clones *grxD* C-terminus in frame with Myc-His tag on plasmid (when used with GrxD_fwd). |
| grxD_PS1 | GTCAGATTACTGCCGTAAAGAAGTTTGAGGAAGCAAGACAgtgtaggctggagctgcttc | For amplifying *cm*^R^ cassette from pKD3 or *kan*^R^ cassette from pKD4. Uppercase nucleotides correspond to region that overlaps with *grxD* while lowercase nucleotides correspond to the region that overlaps into pKD3 and pKD4. |
| grxD_PS2 | GGATGCCCGTTCGCATCCGTCAGTATTGCAGGACGGATTAcatatgaatatcctccttag |  |
| grxD_UP | TTATTTTCCGATGCCGCACTGACCCG | For confirmation of gene deletion by flanking colony PCR |
| grxD_DN | GGCTGGATTGGGGATAGAGAAAGGAC |  |
| hcaT_qPCR_forward | GCTGCTCGGCTTTCTCATCC | For qPCR analysis of reference gene *hcaT* used to calculate ∆Cq values. |
| hcaT_qPCR_reverse | CCAACCACGCTGACCAACC |  |
| SufA_fwd | TAAACATATGGACATGCATTCAGGAACCTTTA | For cloning *sufA* into pET21a |
| SufA_rev | ATAGGGATCCCTATACCCCAAAGCTTTCGCCACAG |  |
